# MLN2238 exerts its anti-tumor effects *via* regulating ROS/JNK/mitochondrial signaling pathways in intrahepatic cholangiocarcinoma

**DOI:** 10.3389/fphar.2022.1040847

**Published:** 2022-10-31

**Authors:** Hao Xu, Guangyu Xu, Qianhui Xu, Chang Xu, Xiaohu Zhou, Yang Bai, Lu Yin, Yuan Ding, Weilin Wang

**Affiliations:** ^1^ Department of Hepatobiliary and Pancreatic Surgery, The Second Affiliated Hospital, Zhejiang University School of Medicine, Hangzhou, China; ^2^ Key Laboratory of Precision Diagnosis and Treatment for Hepatobiliary and Pancreatic Tumor of Zhejiang Province, Hangzhou, China; ^3^ Research Center of Diagnosis and Treatment Technology for Hepatocellular Carcinoma of Zhejiang Province, Hangzhou, China; ^4^ Clinical Medicine Innovation Center of Precision Diagnosis and Treatment for Hepatobiliary and Pancreatic Disease of Zhejiang University, Hangzhou, China; ^5^ Clinical Research Center of Hepatobiliary and Pancreatic Diseases of Zhejiang Province, Hangzhou, China; ^6^ Cancer Center, Zhejiang University, Hangzhou, China; ^7^ Department of Pathology, Affiliated Hangzhou First People’s Hospital, Zhejiang University School of Medicine, Hangzhou, China

**Keywords:** intrahepatic cholangiocarcinoma, proteasome inhibitors, autophagy, apoptosis, ROS/JNK/mitochondrial signaling pathway

## Abstract

**Background:** Intrahepatic Cholangiocarcinoma (iCCA) is a highly malignant tumor with limited treatment options that contributes largely to cancer-related deaths worldwide. Compared with traditional transcriptomic analysis, single-cell RNA sequencing (scRNA-seq) is emerging as a more advanced and popular tool for the in-depth exploration of cellular diversity and molecular complexity. As a next-generation proteasome inhibitor, MLN2238 presents better pharmacodynamics, pharmacokinetics, and therapeutic responses in various cancers. However, its effects and mechanisms of action in iCCA remain unknown.

**Methods:** iCCA tumor heterogeneity was determined based on 4,239 qualified scRNA-seq data from 10 iCCA samples. The potential biological roles of proteasome-related genes in iCCA were investigated using a pseudo-trajectory reconstruction. The effect of MLN2238 on iCCA cell proliferation was estimated using the CCK-8, EdU, and clone formation assays. Flow cytometry was used to examine the effect of added MLN2238 on cell cycle and apoptosis levels. Autophagic flux was detected using AdPlus-mCherry-GFP-LC3B cells. ROS levels and mitochondrial membrane potential were determined using DCFH-DA probing and JC-1 staining. JNK activation and mitochondrial apoptosis were observed using western blotting and immunofluorescence microscopy, respectively. Finally, we used a tumor-bearing mouse model to validate its efficacy *in vivo* for iCCA treatment.

**Results:** Proteasome-related genes were dysregulated in iCCA progression and expressed at higher levels in tumor tissues. MLN2238 suppressed cell proliferation, blocked the cell cycle in the G2/M phase, promoted apoptosis, and induced cytoprotective autophagy in iCCA cells. Furthermore, MLN2238 increased ROS levels and activated the JNK signaling pathway. Inhibition of ROS and JNK activation by NAC and SP600125 significantly reversed MLN2238-induced apoptosis. MLN2238 also suppressed the growth of iCCA tumors *in vivo*.

**Conclusion:** Proteasome-related genes play pivotal roles in iCCA development. MLN2238, as a proteasome inhibitor, induces apoptosis in iCCA cells through ROS/JNK/mitochondrial signaling pathways, and hence, making MLN2238 a potential therapeutic choice for iCCA.

## 1 Introduction

Cholangiocarcinoma (CCA) is a highly malignant tumor that can arise anywhere in the biliary tree (Valle et al., 2021). According to anatomic location, it can be further divided into intrahepatic, perihepatic, and distal CCA (Brindley et al., 2021). Among the three subtypes, intrahepatic CCA (iCCA) takes place in the proximal end of the secondary bile ducts in liver parenchyma, accounting for approximately 15% of primary liver cancers and 3% of gastrointestinal tumors. The incidence and mortality of iCCA have increased worldwide over the past few decades (Banales et al., 2020). Owing to the high degree of aggression and absence of early remarkable symptoms, the prognosis of iCCA is usually poor. Although the majority of patients miss the opportunity for surgery at the first diagnosis, surgery is still the main curative treatment for iCCA (Rizvi et al., 2018). For patients with advanced-stage of iCCA, the combined application of gemcitabine and cisplatin remains the first-line chemotherapy. Recently, some molecularly targeted therapies, such as FGFR and IDH1/IDH2 inhibitors, and immunotherapy, have been shown to possess potential therapeutic effects. However, the mechanisms underlying disease progression and the long-term outcomes of these therapeutic measurements remain uncertain. Consequently, the development of a novel strategy for iCCA treatment is urgently required.

In contrast to traditional transcriptomics, single-cell RNA sequencing (scRNA-seq) can identify specific cell types and provide a unique perspective for studying the regulation, evolution, and interactions of individual cells. In-depth analysis of scRNA-seq enables researchers to uncover the heterogeneity between tumor cells and the complexity of the tumor microenvironment, which are essential for understanding the relevant mechanisms of tumorigenesis and development. Hence, scRNA-seq technology has been increasingly applied in CCA research, both for intrahepatic and distal tumors (Li et al., 2022; Song et al., 2022).

The proteasome, also named “26S proteasome,” consists of a 20S proteolytically active core particle and one or two 19S capped regulatory particles (Adams, 2003). The ubiquitin-proteasome system plays an essential role in the selective degradation of intracellular proteins and, therefore, influences many key cellular biological processes, including cell proliferation, cell cycle, apoptosis, and signal transduction. Several studies have revealed that excessive proteasome activation is correlated with many pathological states, such as cancer, neurodegenerative diseases, and inflammation (Adams, 2004a; Rubinsztein, 2006; Schmidt et al., 2010). In cancer, proteasome inhibition can induce tumor apoptosis and enhance sensitivity to chemotherapy and radiotherapy by altering the balance between proapoptotic and antiapoptotic signaling pathways (Adams, 2004b).

Bortezomib is a first-generation proteasome inhibitor that has been approved for the treatment of multiple myeloma and some hematologic tumors. Although bortezomib exerts anticancer effects on solid tumor malignancies in cell cultures, a single-agent phase II trial reported that bortezomib has limited clinical activity in patients with advanced CCA (Denlinger et al., 2014). In contrast to bortezomib, MLN2238 (ixazomib) is a second-generation proteasome inhibitor that can be administered orally. Furthermore, MLN2238 showed better therapeutic efficacy than bortezomib in both solid and hematologic tumor models (Kupperman et al., 2010). Recent studies have shown that MLN2238 has an encouraging antitumor effect on hepatocellular (Augello et al., 2018) and colon carcinomas (Engür and Dikmen, 2017), osteosarcoma (Liu et al., 2017), and melanoma (Suarez-Kelly et al., 2016). However, the precise antineoplastic mechanism of MLN2238 in iCCA has not yet been demonstrated.

In this study, we included 4,239 qualified scRNA-seq data from 10 iCCA lesions, analyzed tumor heterogeneity, and reconstructed the pseudo-trajectory of malignant cells to investigate the potential biological roles of proteasome-related genes in iCCA. Subsequently, we selected MLN2238 as a therapeutic drug, calculated its IC_50_ in iCCA cells, and observed its effects on cell proliferation, the cell cycle, apoptosis, and autophagy. Furthermore, we have found that MLN2238 induces apoptosis through the ROS/JNK/mitochondrial signaling pathway in iCCA cells. We have also explored the potential therapeutic effect of MLN2238 on iCCA *in vivo* and *in vitro* with the aim of developing a novel strategy for the treatment of the disease.

## 2 Materials and methods

### 2.1 Sources of scRNA-seq datasets

The scRNA-seq data of GSE125449 were acquired from the Gene Expression Omnibus database, and 10 iCCA biopsies were selected for in-depth follow-up analysis. Specific information, including the scRNA-seq gene-barcode matrix, feature data, and UMI count tables of barcodes, has been presented by Ma et al. (Ma et al., 2019).

### 2.2 Quality control and the reduction of dimensionality

The Read10X function from the Seurat toolkit was used to import the above data for iCCA, and the CreateSeuratObject function was used to establish the Seurat object for subsequent analysis. Gene-cell matrices was screened to exclude unqualified cells (<500 transcripts/cells, >5% mitochondrial genes) and genes (<1,000 cells/gene and >20,000 cells/gene). We then employed the NormalizeData function to convert the gene expression into a natural logarithm. The top 2000 highly variable genes were identified for principal component analysis (PCA) *via* the FindVariableFeatures function. Subsequently, FindNeighbors and FindClusters functions were performed to calculate the dimensionality reduction coordinates and unsupervised cell clustering. Single-cell clustering was visualized using the RunUMAP function. Ultimately, the DoubletFinder package was used to remove double cells.

### 2.3 The annotation of cell clustering

First, the FindAllMarkers function from the Seurat toolkit was employed to distinguish cluster-specific marker genes using the default non-parametric Wilcoxon rank sum test with Bonferroni correction. Finally, according to cell localization, marker database, and renowned cell markers from other studies, different cell clusters were annotated, including epithelial cells, T cells, myeloid cells, fibroblasts, B cells, and endothelial cells.

### 2.4 The evaluation of scRNA-seq copy number variation

The CNV of each cell was evaluated using the InferCNV package (https://github.com/broadinstitute/inferCNV/wiki) (Patel et al., 2014). We used immune cells as the reference, and the parameter “cutoff” was set at a value of 0.1. Malignant cells were defined as cells with a CNV score > 0.0005, whereas non-malignant cells had a CNV score < 0.0005.

### 2.5 The single-cell trajectory construction in iCCA

The Monocle2 R package was employed to construct the single-cell trajectory, with the hypothesis that one-dimensional “time” could present high-dimensional expression values to uncover the transition of cell states (Trapnell et al., 2014). Malignant cells were identified and introduced into the R environment. We then used the newCellDataSet function to establish the object (expression Family = negbinomial.size). These malignant cells were sorted in pseudo-time order *via* highly variable genes fitting mean_expression ≥ 0.1 and dispersion_empirical ≥1*dispersion_fit. Furthermore, the reduceDimension () function (reduction_method = “DDRTree” and max_components = 2) was executed for dimensionality reduction and the plot_cell_trajectory function was applied to draw the minimum spanning tree plot. Finally, the related gene changes during the pseudo-time process were calculated using the differentialGeneTest function and are presented as the plot_pesudotime_heatmap.

### 2.6 Bulk RNA-seq data collection and pretreatment

Bulk RNA-seq transcriptomic and clinical data, including eight normal bile ductal tissues and 33 iCCA samples, were downloaded from the Cancer Genome Atlas database (TCGA, https://cancergenome.nih.gov). The gene expression patterns of TCGA-CHOL samples were transformed from Fragments Per Kilo-downloaded Million (FPKMs) into Transcripts Per Kilobase Million (TPMs).

### 2.7 Differential expression of proteasome-related genes

Differentially expressed proteasome-related genes between iCCA and normal tissues were identified using the R software limma package, which met the thresholds of logFC (fold change) > 0.1 and FDR (false discovery rate) < 0.05. The GeomSplitViolin, pheatmap, and ggplot2 packages were used to draw the half-violin, heatmap, and volcano plots.

### 2.8 Chemicals and antibodies

The materials used in this study were MLN2238 (cat. no. S2180, Selleck), chloroquine (CQ, cat. no. HY-17589A, MedChemExpress), N-Acetyl-L-Cysteine (NAC, cat. no. A7250, Sigma-Aldrich), SP600125 (cat. no. HY-12041, MedChemExpress), primary antibodies against LC3B (cat. no. AF4650, Affinity), P62 (cat. no. ab109012, Abcam, Cambridge, United Kingdom), GAPDH (cat. no. 10494-1-AP, Proteintech), (c-J N-terminal kinase JNK (cat. no. 92525, Cell Signaling Technology), phospho-JNK (Thr183/Tyr185) (cat. no. 4668S, Cell Signaling Technology), Bim (cat. no. ab32158, Abcam), cytochrome C (cat. no. ab133504, Abcam), VDAC1/2 (cat. no. 10866-1-AP, Proteintech), PARP1 (cat. no. ab191217, Abcam), Caspase 3 (cat. no. 9662S, Cell Signaling Technology), and Caspase 9 (cat. no. 9502S, Cell Signaling Technology, Danvers, MA). The concentrations of all the primary antibodies used were prepared as recommended by the manufacturer’s instructions.

### 2.9 Cell culture

Two types of human iCCA cell lines, HuCCT-1 and CCLP-1, and one human healthy cholangiocyte cell line HIBEpiC, were preserved in our laboratory. Both iCCA cell lines were cultured in Roswell Park Memorial Institute-1640 (RPMI-1640, cat. no. 01-100-1ACS; Biological Industries) medium supplemented with 10% fetal bovine serum (FBS, cat. no. 10091-148; Gibco) at 37°C with 5% CO_2_. The HIBEpiC cell line was cultured in Minimum Essential Medium (MEM, cat. no. 01-025-5A; Biological Industries) supplemented with 10% FBS at 37°C with 5% CO_2_.

### 2.10 Cell counting kit-8 assay

CCK-8 (cat. no. C0039) were purchased from Beyotime Biotechnology. For concentration-dependent cell viability analysis, HuCCT-1 and CCLP-1 cells were seeded into 96-well plates at a density of 4×10^3^ cells per well for 24 h and then treated with various concentrations of MLN2238 for 24 or 48 h. Subsequently, 10 μL CCK-8 were added into each well and incubated at 37°C for 30 min to 4 h. The optical density (OD) at 450 nm was measured using a multifunctional microplate reader (TECAN SPARK, Switzerland). The IC_50_ values of HuCCT-1 and CCLP-1 cells were calculated using the GraphPad Prism 8 software. For the time-dependent cell viability analysis, iCCA cells were seeded into 96-well plates (4×10^3^ cells/well) 8 h prior to treatment with MLN2238 (IC_50_ and 2×IC_50_ concentrations) for 0, 24, 48, and 72 h. The absorbance values (450 nm) were measured after adding 10 μL CCK-8 to each well for 2 h.

### 2.11 Clone formation assay

HuCCT-1 and CCLP-1 cells were seeded into 6-well plates at a density of 1×10^3^ cells per well. After adherence, the cells were treated with the IC_50_ and 2×IC_50_ concentrations of MLN2238 for 24 h and cultured for approximately 2 weeks. Finally, the clone was identified when more than 50 cells took up the crystal violet (cat. No. C0121, Beyotime) stain.

### 2.12 EdU viability assay

HuCCT-1 and CCLP-1 cells were seeded into 6-well plates and treated with IC_50_ and 2×IC_50_ of MLN2238 for 24 h. Then according to the protocol of manufacturer, the Cell-Light EdU Apollo567 *In Vitro* Kit (cat. no. C10310-1, Ribobio) was used to evaluate the effect on cell proliferation. Finally, EdU-labeled iCCA cells and cell nuclei stained with Hoechst33342 were observed using fluorescence microscopy (Leica DMi8, Germany).

### 2.13 Flow cytometry analysis of cell apoptosis

The Annexin V-FITC Apoptosis Detection Kit (cat. no. C1062, Beyotime) was used to evaluate the level of apoptosis. Briefly, two types of iCCA cells were seeded into 6-well plates at a concentration of 2×10^5^ cells per well. After treatment with IC_50_ and 2×IC_50_ concentrations of MLN2238 for 24 h, cells were collected and double-stained with fluorescein isothiocyanate (FITC) and propidium iodide (PI) following the manufacturer’s protocol. Cell apoptosis was detected using a flow cytometer (BD Biosciences Cytometer, United States), and Flowjo software 7.6.1 was used to calculate the apoptosis rate of each group.

### 2.14 Flow cytometry analysis of cell cycle distribution

Cell cycle distribution was assessed using a Cell Cycle and Apoptosis Analysis Kit (cat. no. C1052, Beyotime). HuCCT-1 and CCLP-1 cells were seeded into 6-well plates (2×10^5^ cells/well) and treated with IC_50_ and 2×IC_50_ concentrations of MLN2238 for 24 h after cell adherence. Then iCCA cells were harvested, fixed in 70% ethanol at 4°C overnight. Fixed cells were then stained using PI for 30 min. Finally, cell cycle distribution was examined using flow cytometry and analyzed using the Modifit LT software 3.1.

### 2.15 Immunofluorescence microscopy

For immunofluorescence of LC3B, HuCCT-1 and CCLP-1 cells were seeded into confocal dishes at a density of 5×10^4^ cells per well. After treatment with MLN2238 (IC_50_ concentration) for 24 h, iCCA cells were fixed with 4% paraformaldehyde, treated with 0.5% Triton X-100, washed with PBST, blocked with 10% FBS for 1 h, and incubated with primary rabbit antibody against LC3B (1:300) at 4°C overnight. The following day, fluorochrome-conjugated anti-rabbit IgG (Alexa Fluor® 488, 1:200, cat. no. ab150077, Abcam) was added to the confocal dishes and subsequently incubated for 1 h. Finally, observations were made using a confocal microscopy (LSM900, Zeiss, Germany). For immunofluorescence of cytochrome C, iCCA cells were seeded into confocal dishes at the same density and treated with IC_50_ and 2×IC_50_ concentrations of MLN2238 for 24 h. Mitotracker (cat. no. C1049, Beyotime) was used to label the mitochondria. After being fixed and blocked, primary rabbit antibody against cytochrome C (1:100) was added to the confocal dishes and incubated at 4°C overnight. Following addition of Fluorochrome-conjugated anti-rabbit IgG and DAPI (cat. no. C1006, Beyotime) staining, the location of cytochrome C proteins was observed using confocal microscopy.

### 2.16 Western blotting

Western blotting was performed as previously described (Xu et al., 2019). Briefly, iCCA cells or tissues were collected and dissolved in a mixture of radioimmunoprecipitation assay lysis buffer (cat. no. G2002, Servicebio) and protease inhibitor cocktail (1:100, cat. no. HY-K0010, MedChemExpress) for 1 h. The BCA assay kit (cat. no. 23250, Thermo Fisher Scientific) was used to calculate protein concentration. Subsequently, a protein marker (cat. no. 26616, Thermo Fisher Scientific), and equivalent quantities of protein were loaded to 4–20% SurePAGE Bis-Tris gels (cat. no. M00656 and M00657, GenScript) for electrophoresis (130 V, 60 min) and transferred onto 0.45 μm polyvinylidene fluoride (PVDF) membranes. After blocking with the QuickBlock™ Blocking Buffer (cat. no. P0235, Beyotime), the PVDF membranes were incubated with primary antibodies at appropriate concentration at 4°C overnight. The membranes were then washed with TBST and incubated with secondary antibodies (1:3000, cat. no. SA00001-2, Proteintech) for 1 h at room temperature. Finally, the protein bands were detected using an ECL kit (cat. no. G2014, Servicebio) and a hypersensitive ECL kit (cat. no. G2020, Servicebio).

### 2.17 Detection of autophagy flux

HuCCT-1 and CCLP-1 cells were seeded into a confocal dish (3×10^4^ cells/well) and then incubated with AdPlus-mCherry-GFP-LC3B (cat. no. C3012, Beyotime) at a MOI of 20 for 48 h iCCA cells were subsequently treated with MLN2238 (IC_50_) for 24 h. Finally, autophagic flux was observed using a ZEISS LSM 900 confocal microscope.

### 2.18 Detection of reactive oxygen species

Reactive oxygen species (ROS) assay kit (cat. no. S0033S, Beyotime) was used to determine the ROS accumulation in CCA cells. Briefly, HuCCT-1 and CCLP-1 cells were seeded into 6-well plates 24 h prior to exposure to Rosup (positive control), IC_50_, and 2×IC_50_ concentrations of MLN2238. iCCA cells were then harvested and co-cultured with DCFH-DA (2,7-dichlorodihydrofluorescein diacetate) for 30 min at 37°C. The levels of ROS accumulation were detected using flow cytometry and confocal microscopy.

### 2.19 Detection of mitochondrial membrane potential

The mitochondrial membrane potential assay kit with JC-1 (cat. no. C2006, Beyotime) was used to detect changes in mitochondrial membrane potential. According to the manufacturer’s instructions, the two types of iCCA cells were seeded into 6-well plates and treated with IC_50_ and 2×IC_50_ concentrations of MLN2238. Treated cells were incubated with 1 ml JC-1 staining work solution for 20 min at 37°C and washed twice using JC-1 staining buffer. Finally, red and green fluorescence were determined using confocal microscopy and flow cytometry.

### 2.20 Mitochondria isolation

Isolation of cytoplasmic and mitochondrial proteins was performed using a Cell Mitochondria Isolation Kit (Cat. no. C3601, Beyotime). Following the manufacturer’s protocol, iCCA cells were harvested and incubated with mitochondrial isolation reagents containing 1 mM PMSF kept in an ice bath for 10 min after IC_50_ and 2×IC_50_ treatment-concentrations of MLN2238. Cell suspensions were then homogenized until half of the cells were stained with trypan blue. Finally, mitochondria were separated from the cytoplasm using differential centrifugation.

### 2.21 Detection of caspase-9 and caspase-3 activity

Caspase-9 and Caspase-3 activity was measured using Caspase-9 activity assay kit (cat. no. C1158, Beyotime) and Caspase-3 activity assay kit (cat. no. C1116, Beyotime). Briefly, HuCCT-1 and CCLP-1 cells were collected and lysed after exposure to the IC_50_ and 2×IC_50_ concentrations of MLN2238 for 24 h. The samples were then centrifuged at 18,000 g for 15 min. Afterwards, the mixture of samples, lysis buffer, and Ac-LEHD-pNA (Caspase-9) or Ac-DEVD-pNA (Caspase-3), was added into 96-well plates and cultured at 37°C for 30–60 min (or overnight, if required). Finally, the OD values at 405 nm were measured using a multifunctional microplate reader, and the activities of Caspase-9 and Caspase-3 were calculated using appropriate pNA standard curves.

### 2.22 *In vivo* studies

MLN2238 was dissolved in 10% 2-hydroxypropyl-β-cyclodextrin (cat. no. H108813, Aladdin) at a concentration of 1.4 mg/ml. For the *in vivo* experiments, 15 BALB/c nude mice (4 weeks old weighing 20 g) were fed under SPF conditions. Approximately 3×10^6^ CCLP-1 cells (100 μL) were injected subcutaneously into the right flanks of the mice. When the size of the tumors reached approximately 100 mm^3^, the animals were randomly assigned into three groups (5 mice each). Group 1: received 10% 2-hydroxypropyl-β-cyclodextrin (negative control group); Group 2: received 7 mg/kg MLN2238 (treatment group) as mentioned above; and Group 3: received 14 mg/kg MLN2238 (treatment group) as mentioned above; all the compounds were administered twice a week *via* oral gavage. Tumor volume (volume = length × width^2^/2) and body weight were recorded during the treatment period. Finally, the mice in the three groups were sacrificed 3 weeks after treatment. The serum from each animal was collected for liver and kidney function tests. The heart, liver, spleen, lungs, and kidneys were collected for hematoxylin and eosin (HE) staining. Half of the tumors was fixed in formalin for immunohistochemical analysis [Ki-67 and phospho-JNK (Thr183/Tyr185)], and the other half was frozen in liquid nitrogen for further western blot analysis. All procedures were reviewed and approved by the Institutional Animal Care and Use Committee, Zhejiang Center of Laboratory Animals (Approval No.ZJCLA-IACUC-20020037).

### 2.23 Extraction and purification of cellular total RNA

The HIBEpiC, HuCCT-1 and CCLP-1 cells were detached and gathered from cell culture dishes with the help of trypsin. The cells were lysed and then the total RNA was isolated and purified by an RNA-Quick Purification Kit (cat. no. ES-RN001, Shanghai YISHAN Biotechnology). The concentrations and quality of RNA products were measured by a spectrophotometer (Nanodrop 2000; Thermo Fisher Scientific, United States).

### 2.24 Operation of quantitative real-time PCR

The cDNA of HIBEpiC, HuCCT-1 and CCLP-1 cell lines were reverse transcribed by a HiScript® III RT SuperMix Kit for qPCR (cat. no. RC323-01, Vazyme). The qRT–PCR procedure was completed on a PCR instrument (QuantStudio 7 Flex, Applied Biosystems, United States) using reagents from a TB Green® Premix Ex Taq™ II (Tli RNaseH Plus) Kit (cat. no. RR820A, Takara). The house-keeping gene GAPDH was set as the internal reference. The relative expression levels of the genes were calculated using the 2−ΔΔCt method. The primers were synthesized by Tsingke Biotechnology Co.,Ltd. The specific sequences of primers used were listed in the additional files.

### 2.25 Statistical analysis

All bioinformatics data and figures were processed using R 4.0.3, Python 3.7.0. All experimental data and figures were processed using GraphPad Prism 8. The results are presented as the mean ± standard deviation from at least three independent experiments. Student’s t-test or one-way analysis of variance (ANOVA), followed by Tukey’s post hoc test, was conducted for statistical analyses. A *p* value less than 0.05 indicated a statistically significant difference (*p < 0.05, **p < 0.01).

## 3 Results

### 3.1 Single-cell transcriptomic atlas of iCCA

To uncover the cellular diversity in iCCA, scRNA-seq analysis was conducted on 10 iCCA lesion biopsies. Following the initial quality control (Supplementary Figure S1A,B), qualified cells were included for further analysis. After normalization of gene expression, the top 2,000 highly variable genes were identified using PCA (Supplementary Figure S1C). Subsequently, the principal components (PC), whose estimated *p* value was less than 0.05, were selected (Supplementary Figure S1D,E), and the optimal resolution was determined by the cluster tree algorithm (Supplementary Figure S2A). Then, based on the PC value and optimal resolution, uniform manifold approximation and projection (UMAP) analysis was performed to visualize the segregated cell clusters (Supplementary Figure S2B). We also found inter-tumor heterogeneity among the different iCCA samples (Supplementary Figure S2C). Potential double cells were identified and removed using the DoubletFinder package (Supplementary Figure S2D). Ultimately, 4,239 high-quality cells were obtained for in-depth analysis.

**FIGURE 1:**
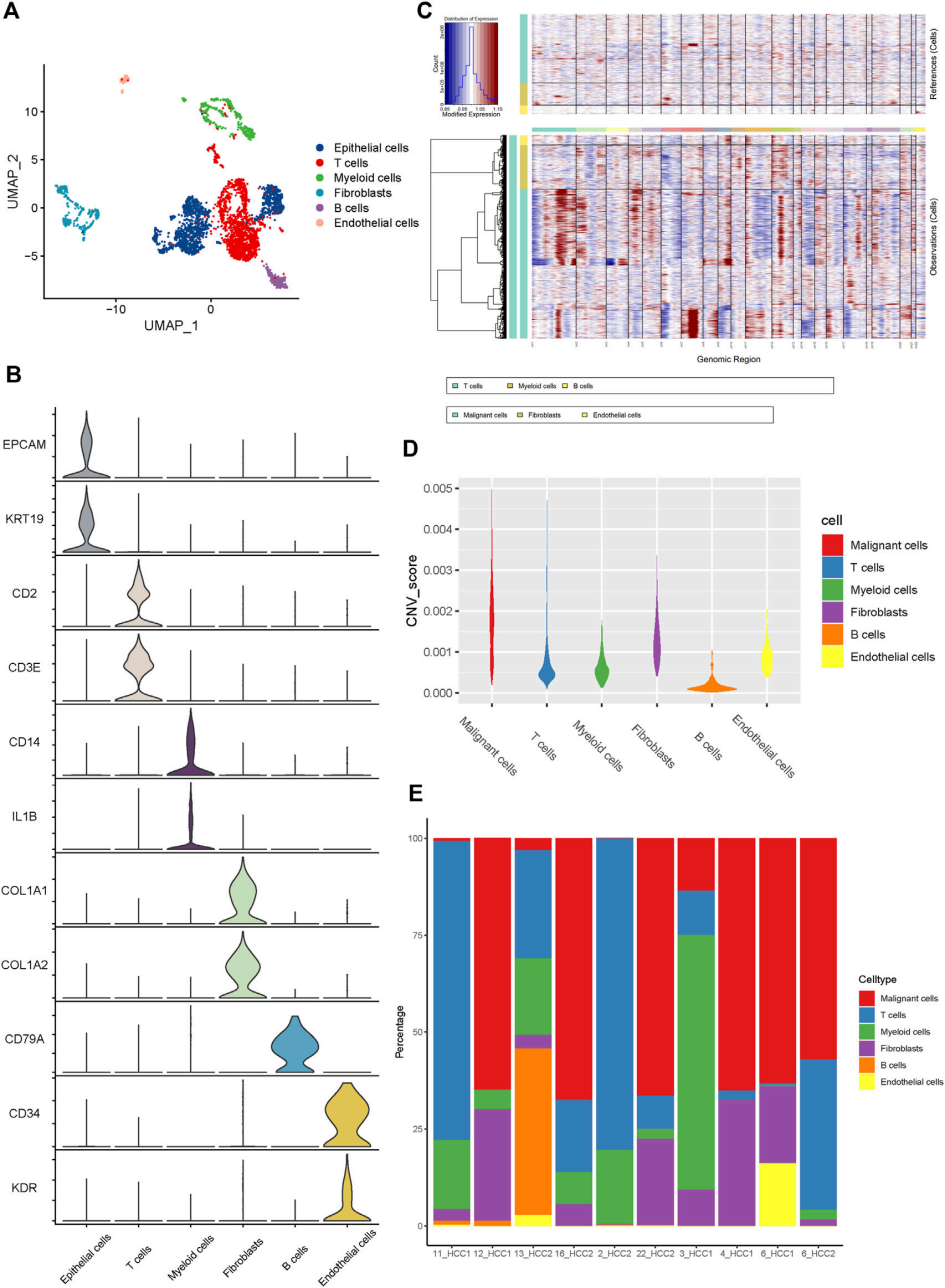
The Landscape of single-cell transcriptomic analysis of iCCA. **(A)** The UMAP plots of the six main cell types from 10 iCCA samples. **(B)** The violin plots showing the expression levels of representative markers across the six main cell types. **(C)** The hierarchical heatmap displaying the large-scale CNVs in iCCA. **(D)** The violin plots revealing the differences of CNV scores among the six main cells types in iCCA. **(E)** The relative percentage of each cell type in 10 iCCA sample.

Six main cell clusters were identified in parallel according to unbiased clustering and typical markers (Figure 1A). These identified cell subgroups were as follows: 1) epithelial cells highly expressing EPCAM and KRT19; 2) T cells characterized by high CD2 and CD3E expression; 3) myeloid cells with high expression of CD14 and IL1B; 4) fibroblasts specifically expressing COL1A1 and COL1A2; 5) B cells with increased expression of CD79A; and 6) endothelial cells specifically expressing CD34 and KDR (Figure 1B and Supplementary Figure S2E).

For further validation of the malignant cells, the chromosomal CNV of each sample was evaluated using the InferCNV package. As presented in Figure 1C, the immune cells, including T cells, myeloid cells, and B cells, were plotted at the top, whereas the parenchymal cells containing epithelial cells, fibroblasts, and endothelial cells were plotted at the bottom. Compared with immune cells (acting as a negative control), epithelial cells were characterized by the amplification and deletion of multiple chromosomes, such as chromosomes 1, 12, and 14 (Figure 1C). In addition, the CNV levels of epithelial cells were significantly higher than those of the fibroblasts, compared to immune cells and endothelial cells which showed lower levels of CNV (Figure 1D). Therefore, we inferred that the previously identified clusters of epithelial cells were the main source of malignant cells in iCCA tissues. It was worth noting that the proportion of cell clusters between iCCA samples varied significantly (Figure 1E), further indicating tumor heterogeneity.

### 3.2 Alteration of proteasome-related genes during iCCA progression

**FIGURE 2:**
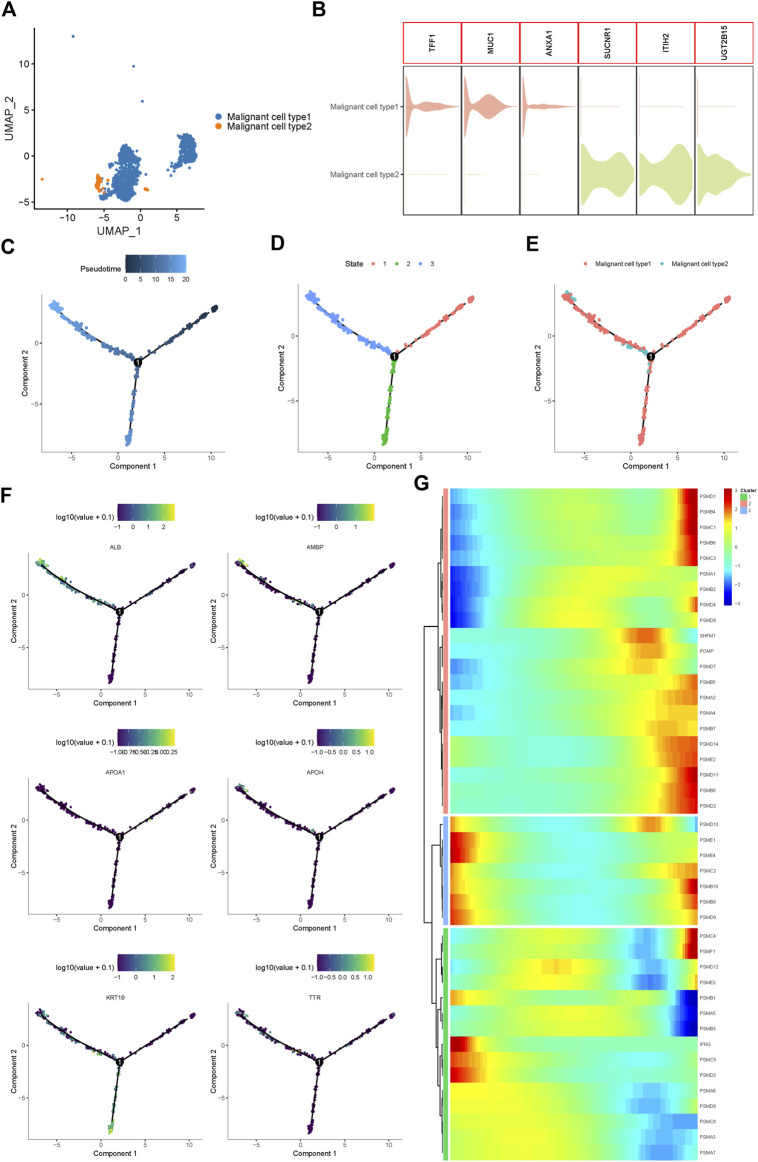
Proteasome-related genes are significantly out of balance during the progression of iCCA cells. **(A)** Two main malignant iCCA cell types were identified by UMAP analysis. **(B)** The violin plots showing the representative markers of the two malignant cell types. **(C–E)** Simulation of the differentiation trajectory of malignant iCCA cell types. **(F)** The distribution of representative genes in pseudo-trajectory of malignant cells of iCCA. **(G)** Heatmap hierarchical clustering showing differentially expressed proteasome-related genes along with the pseudo-time curve.

Using UMAP analysis, two types of malignant cells were identified (Figure 2A). As shown in Figure 2B, malignant cell type 1 specifically expressed TFF1, MUC1, and ANXA1, while type 2 malignant cells specifically expressed SUCNR1, ITIH2, and UGT2B15. To further identify the origins of malignant cells and elucidate the oncogenesis of iCCA, we performed a pseudo-time trajectory analysis. The tree structure of the entire lineage differentiation trajectory was constructed using the monocle2 R package (Figure 2C). Over pseudo-time, the malignant cells experienced three states (Figure 2D): before the branches (State 1) and two different branches (States 2 and 3). Type 1 malignant cells underwent the entire lineage differentiation trajectory, while type 2 malignant cells occurred mainly at the end of one trajectory branch (Figure 2E). Our findings suggest that a part of type 1 malignant cells might be derived from tumors and transform into two different types of malignant cells during the development of iCCA. Consistently, some previously reported genes associated with tumor progression, such as APOH and KRT19, were also gradually upregulated with the development of pseudo-time trajectories (Figure 2F).

Proteasome-related genes play key roles in the formation of proteasomal structures and in maintaining its activity. Recent studies have revealed that some proteasome-related genes, such as PSMA3 and PSMC2, can promote the progression of malignant biliary tumors and act as potential biomarkers to predict prognosis (Verathamjamras et al., 2017; Duan et al., 2021; Zhu et al., 2021). To fully explore the function of the proteasome in the progression of iCCA, 43 proteasome-related genes were included, and the hierarchical heatmap showed that these genes, such as PSMD1, PSMB4, PSMD13, and PSMC4, were out of balance during trajectory development (Figure 2G). In conclusion, significant alterations in the expression of proteasome-related genes in the pseudo-time trajectory indicated that the proteasome system plays an important role in the tumorigenesis and development of iCCA.

### 3.3 Proteasome inhibitor MLN2238 presented its anti-cancer potential on iCCA cells

**FIGURE 3:**
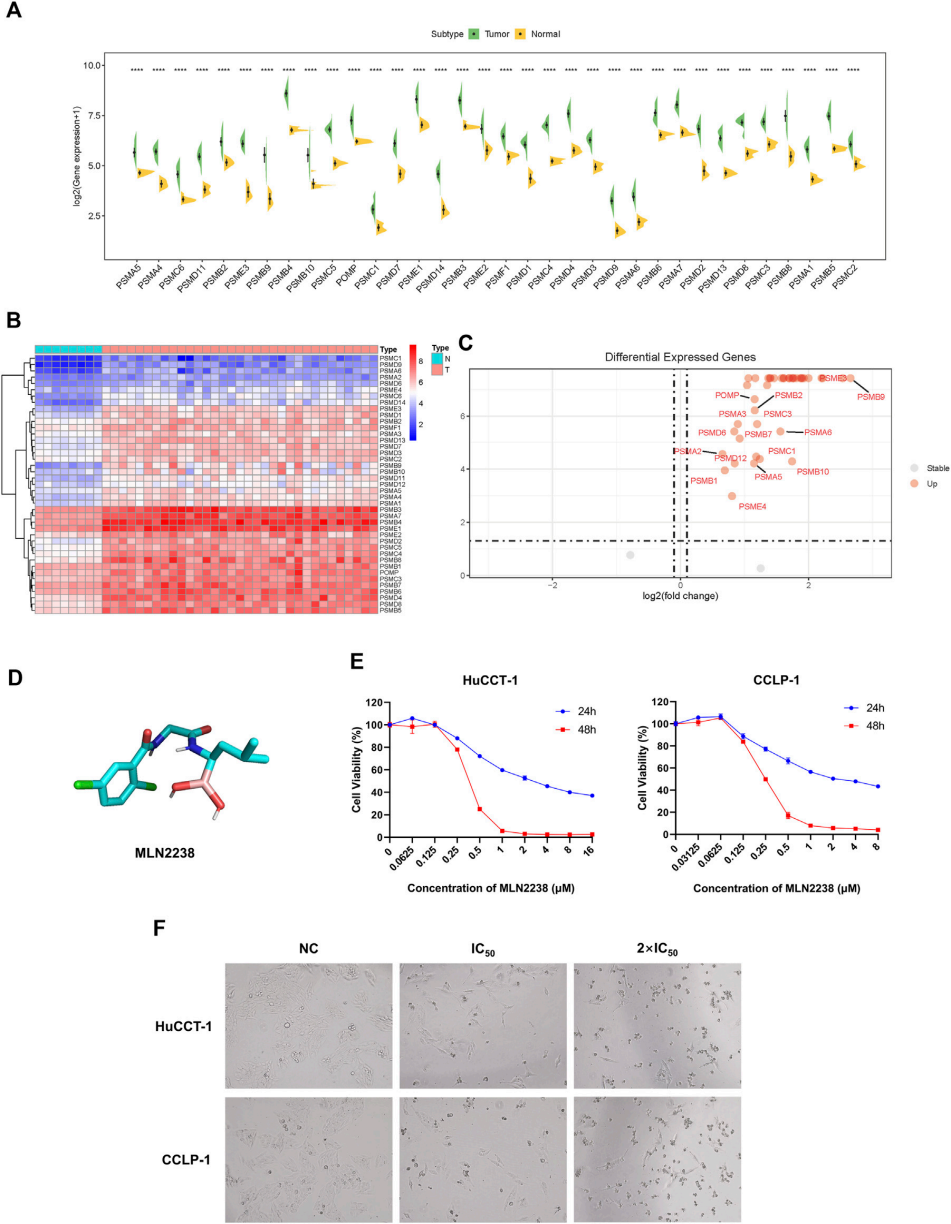
Anti-cancer effect of MLN2238 on iCCA cells. **(A)** The expression levels of proteasome-related genes between normal and iCCA tissues. **(B)** The heatmap showing the proteasome-related genes expression levels in normal and tumor samples. **(C)** The Volcano Plot displaying the distribution of proteasome-related genes between normal tissues and malignant tumor lesions. **(D)** Chemical structures of MLN2238. **(E)** The IC50 values of MLN2238 in iCCA cells (HuCCT-1 and CCLP-1). **(F)** Morphological alteration of HuCCT-1 and CCLP-1 cells after MLN2238 treatment, which observed by microscope (×100 magnification).

To further reveal the expression levels of proteasome-related genes, eight normal tissues and 33 iCCA samples were collected from TCGA. Proteasome-related genes appeared to be significantly more expressed in iCCA samples than in normal tissues (Figure 3A). The heatmap indicated the specific distribution of proteasome-related genes in each sample (Figure 3B), while the volcano map clearly and intuitively identified proteasome-related genes, which exhibited a significant difference in expression levels between normal and iCCA tissues (Figure 3C). We also examined the expression levels of some proteasome-related genes between normal cholangiocyte cell line (HIBEpiC) and two iCCA cell lines (HuCCT-1 and CCLP-1) using qRT-PCR. The result suggested those genes were upregulated in iCCA cell lines (Supplementary Figure S4).Because proteasome-related genes are generally expressed at higher levels in iCCA tissues and cell lines, inhibition of proteasome activity is considered a promising target for the treatment of iCCA.

In view of its better therapeutic efficacy than bortezomib, MLN2238 was chosen as the next generation proteasome inhibitor for subsequent analysis. The chemical structure of MLN2238 is shown in Figure 3D. The viability of each iCCA cell line significantly decreased with the increasing MLN2238 concentration after 24 h or 48 h of drug treatment (Figure 3E). The IC_50_ values for HuCCT-1 and CCLP-1were 3.523 ± 0.012 µM and 2.682 ± 0.080 µM, respectively, after 24 h, and 0.366 ± 0.001 µM and 0.257 ± 0.001 µM, after 48 h, respectively. Morphological alterations were also observed in iCCA cells using optical microscopy after treatment with IC_50_ and 2×IC_50_ concentrations of MLN2238 for 24 h (Figure 3F). The classical apoptotic morphological features, round cells, and detachment from the plates indicated that MLN2238 possessed a potential anti-cancer effect on iCCA cells.

To determine the safety of MLN2238 *in vitro*, we assessed its effect on proliferation and apoptosis in HIBEpiC, HuCCT-1 and CCLP-1 cells. Under the same concentration, HIBEpiC cells displayed higher proportion of EdU-positive cells (Supplementary Figure S5) and lower apoptosis rate (Supplementary Figure S6) compared to iCCA cell lines, indicating normal cholangiocytes were less sensitive to MLN2238.

### 3.4 MLN2238 influence on proliferation, cell cycle, and apoptosis of iCCA cells

**FIGURE 4:**
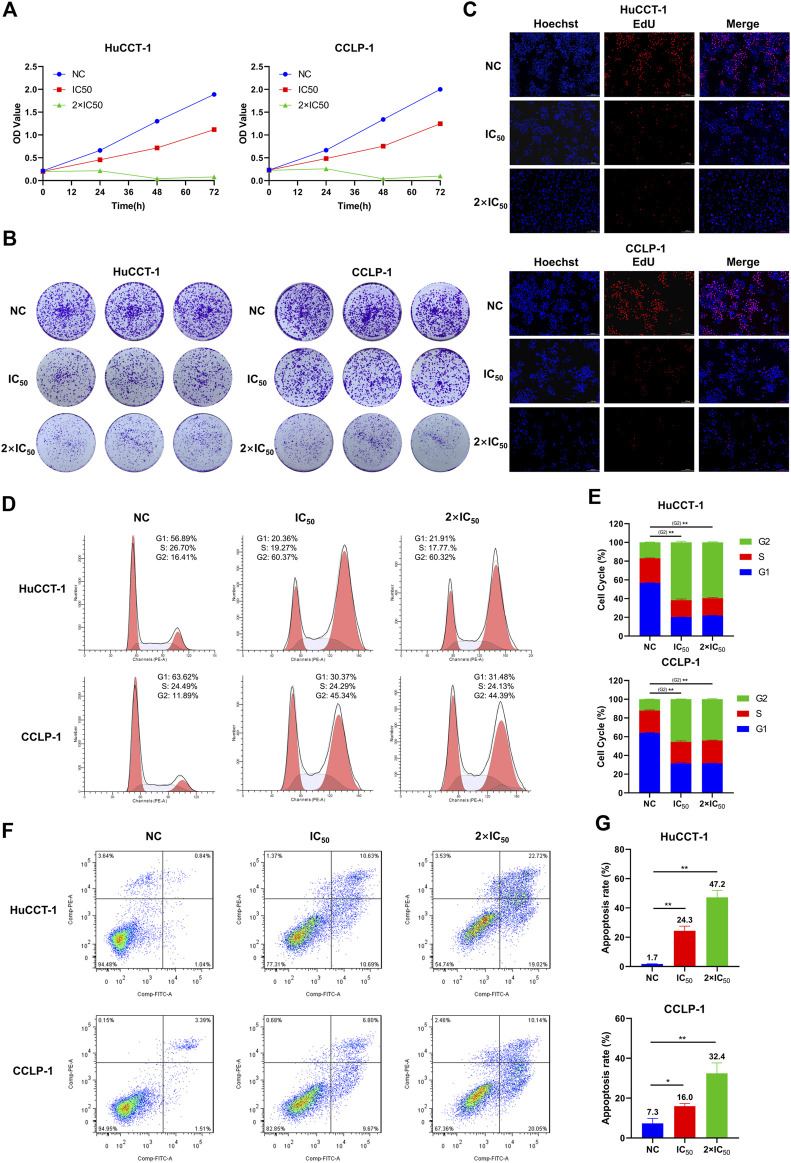
MLN2238 inhibited the proliferation, blocked the cell cycle in the G2/M phase and induced apoptosis in iCCA cells. The proliferation ability of HuCCT-1 and CCLP-1 cells after MLN2238 treatment were evaluated *via* **(A)** cell counting kit-8 analysis, **(B)** clone formation and **(C)** EdU viability assay. **(D)** Cell cycle distribution was detected by flow cytometry analysis after IC50 and 2×IC50 concentration of MLN2238 treatment for 24 h. **(E)** The histogram shows the percentage of HuCCT-1 and CCLP-1 cells in G1-S-G2-phase. **(F)** The apoptotic iCCA cells were measured *via* flow cytometry analysis after treatment with IC50 and 2×IC50 concentration of MLN2238 for 24 h. **(G)** The histogram displays the quantitative analysis of the apoptosis rate of HuCCT-1 and CCLP-1.

To explore the effect of MLN2238 on the proliferative capacity of iCCA cells, a CCK-8 assay was conducted to measure cell viability after IC_50_ and 2×IC_50_ concentrations of MLN2238 treatment for 24, 48, and 72 h. The OD values gradually increased after MLN2238 treatment during 24–72 h, indicating inhibition of cell proliferation in a time-dependent manner (Figure 4A). Consistent with the CCK-8 assay, the clone formation ability (Figure 4B) and proportion of EdU-positive cells (Figure 4C) were significantly reduced after 24 h of MLN2238 treatment (IC_50_ and 2×IC_50_ concentrations).

To further elucidate the inhibitory effect of MLN2238 on iCCA cell proliferation, the cell cycle distribution was evaluated using flow cytometry analysis. After MLN2238 treatment for 24 h, the percentages of HuCCT-1 and CCLP-1 in the G2/M phase were significantly increased, whereas the cell population in the G0/G1 phase showed a decreasing trend (Figures 4D,E). These results indicated that MLN2238 blocked the cell cycle in the G2/M phase.

Based on the typical apoptotic morphological features observed (Figure 3F), we subsequently validated the MLN2238-induced apoptosis-promoting effect using flow cytometry analysis. The apoptosis rates of HuCCT-1 and CCLP-1 were markedly increased in a dose-dependent manner after IC_50_ and 2×IC_50_ concentrations of MLN2238 treatment for 24 h (Figures 4F,G).

### 3.5 MLN2238 induction of autophagy in HuCCT-1 and CCLP-1 cells

**FIGURE 5:**
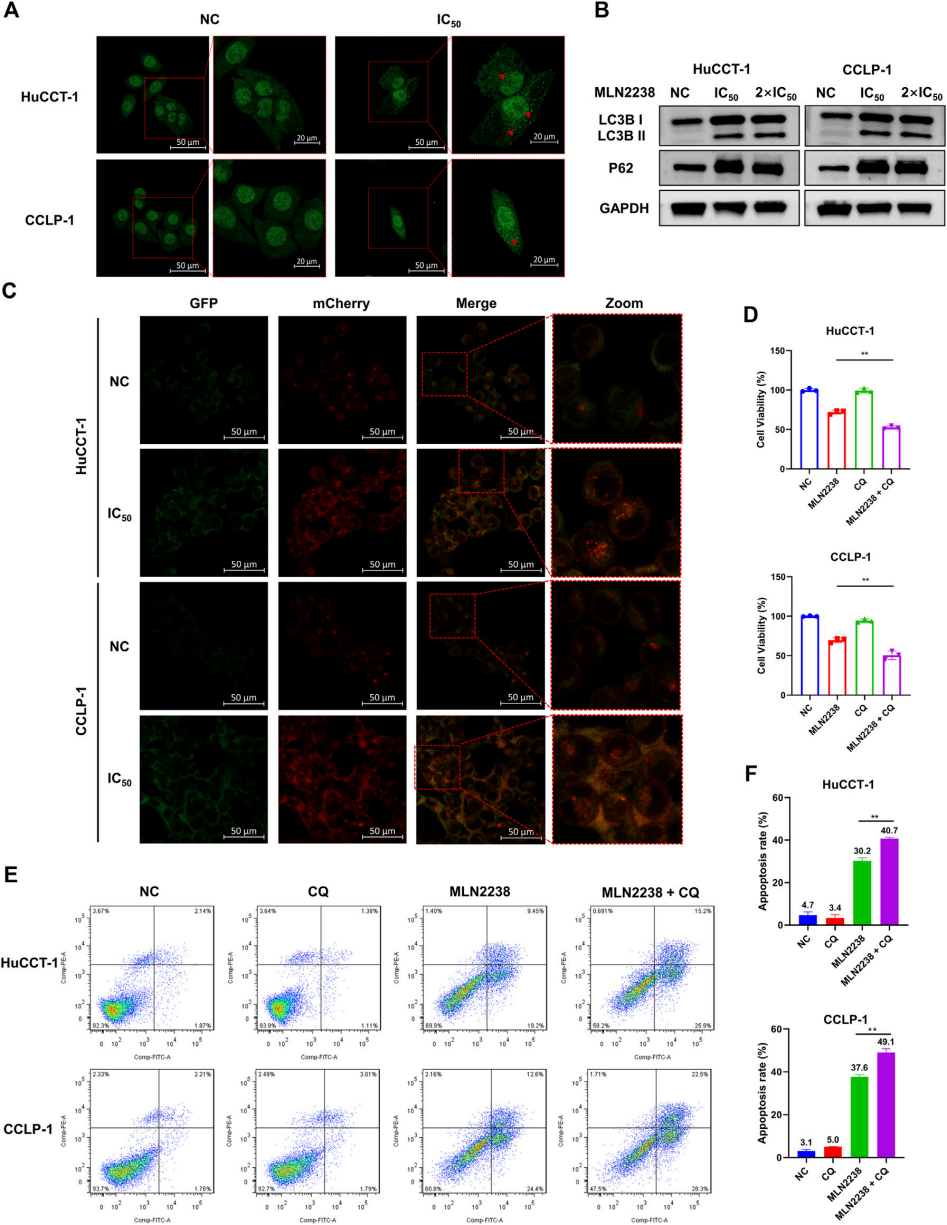
MLN2238 induced the autophagy in HuCCT-1 and CCLP-1 cells. **(A)** Autophagy induction was observed by immunofluorescence microscopy after MLN2238 treatment for 24 h (Red arrows points to LC3B puncta). **(B)** The protein expression levels of LC3B and P62 after treatment with IC50 and 2×IC50 concentration of MLN2238 were analyzed *via* Western Blots. **(C)** Ad-mCherry-GFP-LC3 was used to estimate the autophagy flux after MLN2238 treatment in iCCA cells. **(D)** Cell counting kit-8 analysis and **(E)** flow cytometry analysis was performed to evaluated the cell viability after treatment with MLN2238 and autophagic inhibitor (chloroquine). **(F)** The quantitative apoptosis rates of HuCCT-1 and CCLP-1 cells are presented *via* histogram.

The ubiquitin-proteasome system and autophagy are the two main pathways for intracellular protein degradation. Several studies have shown that proteasome inhibition promotes cytoprotective autophagy (Zhu et al., 2010; Zang et al., 2012). To further investigate the exact effect of MLN2238 on autophagy in iCCA cells, immunofluorescence microscopy and western blotting were performed. The LC3B-positive, punctate dots (indicated by red arrows) (Figure 5A), in HuCCT-1 and CCLP-1 dramatically increased after exposure to the IC_50_ concentration of MLN2238 for 24 h. Western blot analysis showed that the expression of LC3B II significantly increased after MLN2238 treatment (Figure 5B). Interestingly, the protein expression levels of P62 in iCCA cells were also markedly increased, which is related to the inhibitory effect of MLN2238 on proteasomal activity, consistent with previous studies (Sha et al., 2018). To clearly determine whether MLN2238 induces autophagy or blocks autophagy flux in the late phase, AdPlus-mCherry-GFP-LC3B was transfected into HuCCT-1 and CCLP-1 cells and observed using confocal microscopy. As shown in Figure 5C, red dots were obviously increased after IC_50_ concentration of MLN2238 treatment for 24 h, indicating the drug induction in both cell types.

Our previous data showed that MLN2238 could promote apoptosis in iCCA cells. To further explore the relationship between autophagy and apoptosis induced by MLN2238, a classical autophagy inhibitor, CQ, was used to block autophagic flux. The combination of MLN2238 (IC_50_ concentration) with 10 μM CQ for 24 h resulted in higher viability reduction in HuCCT-1 and CCLP-1 cells compared to MLN2238 alone, and a single dose of 10 μM CQ seemed to have no obvious cytotoxicity (Figure 5D). Flow cytometry analysis employing FITC and PI staining also showed that the combined treatment (IC_50_ concentration MLN2238 and 10 μM CQ) induced a higher apoptosis rate than MLN2238 treatment alone (Figures 5E,F). Our findings demonstrated that MLN2238 induced autophagy, and that inhibition of autophagy could enhance the MLN2238-induced pro-apoptotic effect in iCCA cells. Therefore, autophagy may act as a protective mechanism following MLN2238 administration.

### 3.6 MLN2238 induction of iCCA cell apoptosis *via* ROS/JNK/mitochondrial pathways

**FIGURE 6:**
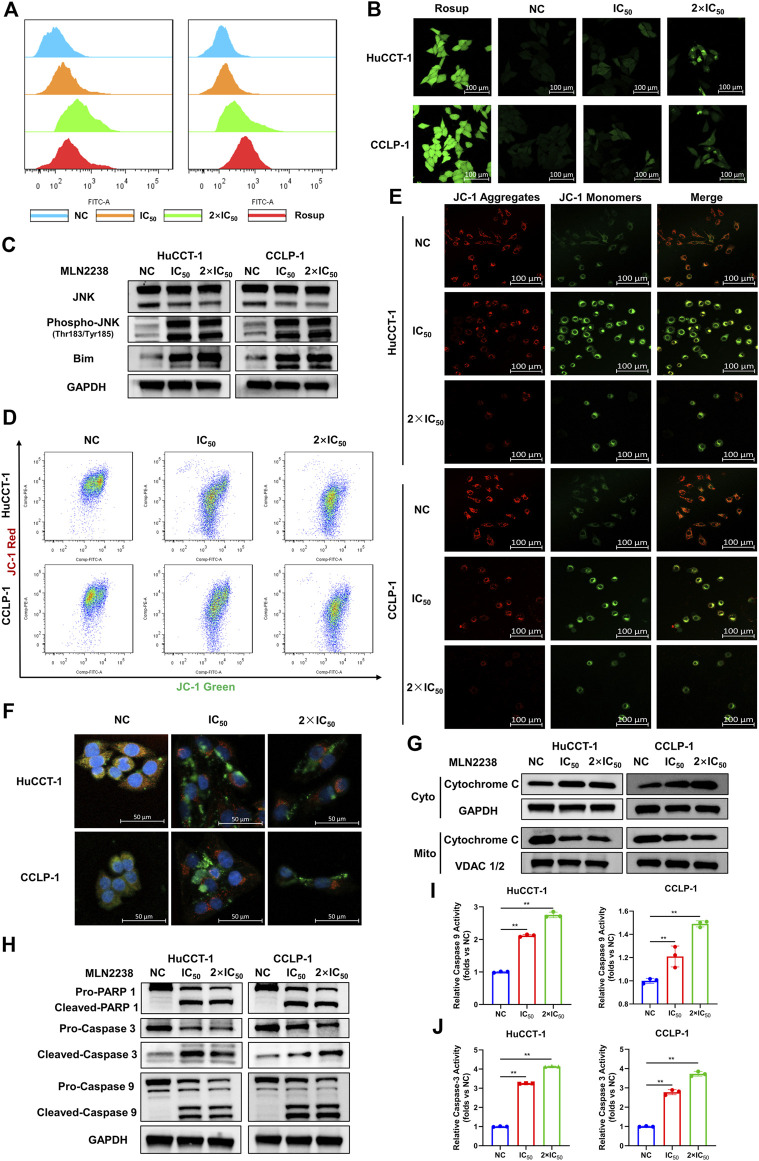
MLN2238 induced apoptosis via ROS/JNK/Mitochondrial signaling pathways in HuCCT-1 and CCLP-1 cells. ROS levels of iCCA cells after MLN2238 treatment (IC50 and 2×IC50 concentration, Rosup acted as active control) for 24 h were measured *via* **(A)** flow cytometry and **(B)** confocal microscopy. **(C)** JNK, phospho-JNK(Thr183/Tyr185) and Bim protein expression levels were analyzed by Western Blots. The changes of mitochondrial membrane potential after IC50 and 2×IC50 concentration MLN2238 treatment were evaluated by **(D)** flow cytometry and **(E)** confocal microscopy. **(F)** The distribution of Cytochrome C after MLN2238 treatment with IC50 and 2×IC50 concentration was observed *via* confocal microscopy (red fluorescence indicated mitochondria, green fluorescence indicated Cytochrome C protein and blue fluorescence indicated nucleus). **(G)** The protein expression of Cytochrome C in cytoplasm and mitochondria after MLN2238 treatment for 24 h was determined *via* Western Blotting. **(H)** The apoptotic-related protein expression levels after IC50 and 2×IC50 concentration MLN2238 treatment were detected by Western Blots. The histograms show the **(I)** Caspase-3 and **(J)** caspase-9 activity of HuCCT-1 and CCLP-1 cells treated with specific MLN2238 concentrations.

Depending on its concentration, ROS play a contradictory role in tumor progression, either by promoting oncogenesis or inducing cell apoptosis (Hayes et al., 2020). Excessive ROS production usually causes oxidative stress and participates in different types of cell death. Several studies have revealed that proteasome inhibitors can mediate oxidative stress, which plays an essential role in apoptosis (Fan et al., 2011). However, the exact relationship between MLN2238 and ROS production in cancer is still unknown. ROS levels were significantly increased after IC_50_ and 2×IC_50_ concentrations of MLN2238 treatment for 24 h, as detected by flow cytometry and confocal microscopy analysis of DCFH-DA co-culture (Figures 6A,B and Supplementary Figure S3A). Several studies have shown that ROS can modulate activation of the JNK pathway to promote apoptosis in tumors (Shan et al., 2016; Wang et al., 2020). Western blotting results showed that, compared with JNK, the expression of phospho-JNK (Thr183/Tyr185) was significantly increased in both HuCCT-1 and CCLP-1 cells, indicating that MLN2238 possibly activated the ROS/JNK pathway (Figure 6C). Furthermore, we found that Bim expression was higher after MLN2238 treatment for 24 h (Figure 6C).

The JC-1 fluorescence probe was used to detect changes in mitochondrial membrane potentials, which convert JC-1 aggregates (red fluorescence) to JC-1 monomers (green fluorescence) during the process of mitochondrial membrane potential reduction. After 24-h MLN2238 treatment, JC-1 green signals were significantly increased, while JC-1 red signals were decreased (Figure 6D). The ratio of green-to-red fluorescence was dramatically elevated in both types of iCCA cells (Supplementary Figure S3B). Consistent with the flow cytometry analysis, the images obtained using confocal microscopy also demonstrated the conversion from red to green fluorescence (Figure 6E). Flow cytometry and confocal microscopy analyses demonstrated that MLN2238 reduced the mitochondrial membrane potential in HuCCT-1 and CCLP-1 cells.

The alteration of the mitochondrial membrane potential can subsequently trigger the release of cytochrome C. As indicated in Figure 6F, the mitochondria were labeled with Mitotracker (red fluorescence), the cytochrome C proteins were marked by immunofluorescence (green fluorescence), and the cell nuclei were stained using DAPI (blue fluorescence). Compared with the NC group, the red and green fluorescence of MLN2238-treated groups were distinctly separated, which indicated that MLN2238 promoted the translocation of cytochrome C from the mitochondria to the cytoplasm. With the help of cell mitochondria isolation, we further validated that the expression of cytoplasmic cytochrome C was markedly increased, whereas mitochondrial cytochrome C expression was dramatically decreased after treatment with IC_50_ and 2×IC_50_ concentrations of MLN2238 (Figure 6G). Subsequently, downstream Caspase-9, Caspase-3, and PARP-1 were detected using western blotting. The cleaved forms of all three proteins were expressed at high levels in HuCCT-1 and CCLP-1 cells after MLN2238 treatment (Figure 6H). Caspase-9 and Caspase-3 activity assay also demonstrated that MLN2238 activated both enzymes, which are closely associated with the mitochondria-related apoptotic pathway (Figures 6I,J).

### 3.7 Inhibition of ROS and JNK activation *versus* MLN2238-induced apoptosis in iCCA cells

**FIGURE 7:**
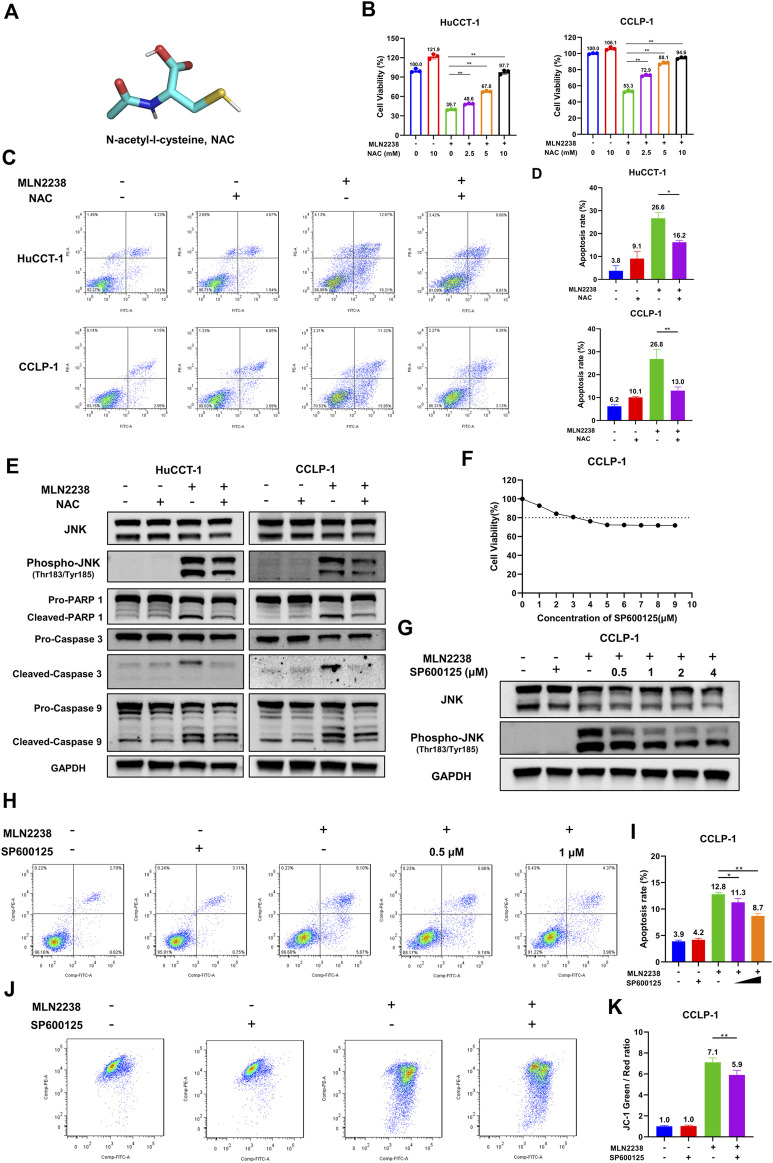
Inhibition ROS and JNK activation reversed the apoptosis induced by MLN2238 in iCCA cells. **(A)** Chemical structures of N-Acety-L-Cysteine (NAC). **(B)** The effect of MLN2238 on cell viability of HuCCT-1 and CCLP-1 cells were reversed by NAC. **(C)** NAC reversed the apoptosis of iCCA cells induced by MLN2238 treatment. **(D)** The histograms display the apoptosis rates of MLN2238 and NAC treatment. **(E)** The expression levels of JNK, phospho-JNK(Thr183/Tyr185) and apoptotic-related protein after IC50 concentration MLN2238 and 10 mM NAC treatment were analyzed *via* Western Blotting. **(F)** CCLP-1 cell was treated with various concentrations of SP600125 for 24 h, and cell counting kit-8 was used to measure the cell viability. **(G)** Western Blots was performed to detected the JNK and phospho-JNK(Thr183/Tyr185) protein expression levels after MLN2238 and SP600125 treatment. **(H)** CCLP-1 cells were preincubated with 0.5 μM or 1 μM SP600125 for 2 h and then treated with IC50 concentration MLN2238 for 24 h. The apoptosis was detected by flow cytometry. **(I)** The quantitative analysis of apoptosis rate of CCLP-1 cells after MLN2238 and SP600125 treatment. **(J)** SP600125 reversed the changes of mitochondrial membrane potential induced by MLN2238. 1 μM SP600125 was pretreated for 2 h and then co-incubated with MLN2238 (IC50 concentration) for 24 h. **(K)** The quantitative analysis of JC-1 green and red fluorescence ratio after MLN2238 and SP600125 treatment.

To further confirm the mechanism of MLN2238-induced apoptosis in iCCA cells, the cells were pretreated with the classical antioxidant NAC (Figure 7A) for 2 h before the addition of MLN2238. First, confocal microscopy analysis confirmed that NAC, acting as an ROS scavenger, diminished the intracellular ROS levels induced by the IC_50_ concentration of MLN2238 (Supplementary Figure S3C). The effect of MLN2238 on cell viability was gradually reversed with increasing NAC concentration from 2.5 mM to 10 mM in HuCCT-1 and CCLP-1 cells (Figure 7B). Similar to the CCK-8 assay, apoptotic flow cytometry analysis illustrated that 10 mM NAC treatment significantly reduced the apoptosis rates induced by 24-h exposure to MLN2238 (Figures 7C,D). We also discovered that, compared with MLN2238 alone, the combined application of MLN2238 and NAC markedly decreased the expression levels of phospho-JNK (Thr183/Tyr185), which further verified that ROS could act upstream to activate the JNK signaling pathway in iCCA cells (Figure 7E). In addition, the expression of cleaved Caspase-9, Caspase-3, and PARP-1 was reversed by treatment with 10 mM NAC (Figure 7E).

SP600125 is regarded as a typical JNK inhibitor that suppresses JNK activation. However, several studies have revealed that SP600125 is cytotoxic and can induce apoptosis in cancer cells (Xia et al., 2006). The CCK-8 assay demonstrated that treatment with more than 3 μM SP600125 for 24 h reduced the viability of CCLP-1 cells (cell viability ≤ 80%) (Figure 7F). Western blotting was performed to validate the inhibitory effect of SP600125 on JNK activation. SP600125 reversed the MLN2238-induced increase in phospho-JNK (Thr183/Tyr185) expression in a dose-dependent manner (Figure 7G). To balance cytotoxicity and sufficient JNK activation inhibitory effects, the cells were pretreated with 0.5 μM or 1 μM SP600125 for 2 h before IC_50_ concentration MLN2238 treatment. In contrast to the MLN2238 group, the combination treatment of MLN2238 with SP600125 significantly diminished the apoptosis rates in iCCA cells, indicating that the activation of JNK played a key role in MLN2238-induced apoptosis (Figures 7H,I). Flow cytometry analysis using JC-1 staining also revealed that the MLN2238-induced reduction in mitochondrial membrane potential was markedly reversed by 1 μM SP600125 treatment (Figures 7J,K). Inhibition of JNK activation has been shown to reduce iCCA cell apoptosis, possibly by influencing the mitochondria-related apoptosis pathway.

### 3.8 MLN2238 effect on tumor growth in iCCA xenograft model

**FIGURE 8:**
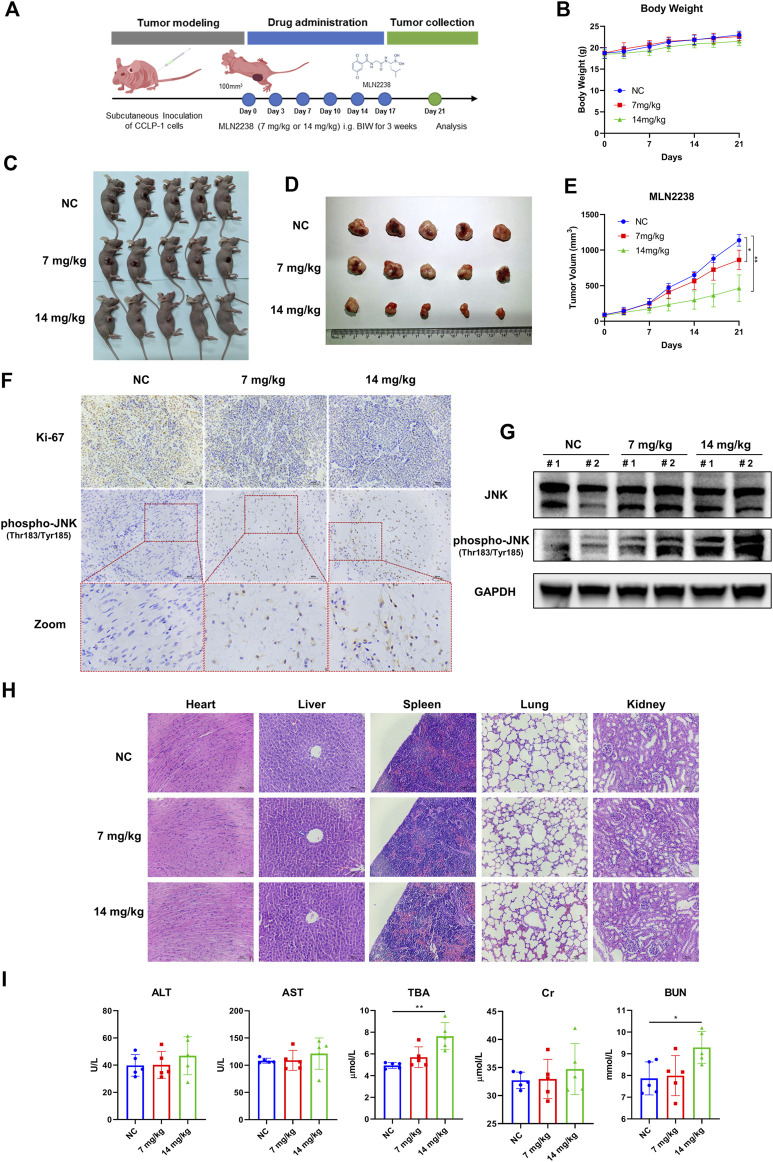
MLN2238 suppressed the tumor growth in the xenograft model of iCCA. **(A)** The scheme of iCCA xenograft model construction and MLN2238 administration *in vivo*. **(B)** The body weight of tumor-bearing mice after MLN2238 treatment during the experiment. **(C,D)** The images show the unresected and resected xenograft iCCA for each group. **(E)** The tumor volumes after MLN2238 (7 mg/kg or 14 mg/kg) treatment were calculated twice a week during the experiment. **(F)** The Ki-67 and immunohistochemistry assays of phospho-JNK(Thr183/Tyr185) for xenograft iCCA. **(G)** The protein expression levels of JNK and phospho-JNK(Thr183/Tyr185) in tumor tissues were analyzed *via* Western Blotting. **(H)** The H&E stains of heart, liver, spleen, lung and kidney tissues of tumor-bearing mice after different MLN2238 concentrations treatment were used to evaluate the biosafety. **(I)** The serum ALT, AST, TBA, Cr and BUN were detected after MLN2238 treatment at the end of the experiment.

To investigate the potential therapeutic effect of MLN2238 *in vivo*, a nude mouse model bearing a subcutaneous iCCA xenograft was established. A dose of 7 mg/kg or 14 mg/kg of MLN2238 was dissolved in 10% 2-hydroxypropyl-β-cyclodextrin and administered twice a week for 3 weeks (Figure 8A). The body weight of mice is usually used to evaluate drug toxicity. There was no significant difference in mice body weight among the three groups after MLN2238 treatment (Figure 8B). Compared with the control group, administration of 7 mg/kg or 14 mg/kg MLN2238 markedly reduced tumor size and volume (Figures 8C–E). Immunohistochemical analysis also demonstrated that treatment with MLN2238 significantly decreased the expression of the cell proliferation marker ki-67 and increased the expression of phospho-JNK (Thr183/Tyr185) in tumor tissues (Figure 8F). Western blotting analysis revealed that MLN2238 induced JNK activation in a dose-dependent manner (Figure 8G), confirming the results obtained from immunohistochemistry experiments. To assess the biological safety of MLN2238 *in vivo*, H&E-stained images of the mouse heart, liver, spleen, lung, and kidney were observed using optical microscopy (Figure 8H). After treatment with 7 or 14 mg/kg MLN2238 for 3 weeks, there was no severe damage to important tissues and organs. Although the blood tests of mice indicated an increased expression of TBA and BUN after high concentration administration of MLN2238; no significant difference has been noted for ALT, AST, and Cr levels among the three groups (Figure 8I). In conclusion, MLN2238 possessed relatively good biological safety and suppressed iCCA tumor growth *in vivo*.

## 4 Discussion

iCCA is a highly aggressive malignant cancer derived from biliary epithelial cells, with high malignancy and poor prognosis. However, treatment options for iCCA remain limited. To date, surgical resection is still the preferred curative method, but the postoperative recurrence rate remains high. Although treatment options for iCCA have been extended to adjuvant therapy, liver transplantation, FGRF2 molecular therapy, and immunotherapy, the overall survival rate remains unsatisfactory (Clements et al., 2020). Consequently, overwhelming research has been conducted to develop special treatments for iCCA. However, over the past few decades, the subsequent analysis of nearly all iCCA samples has been based on traditional RNA sequencing data, which limits the in-depth exploration of cellular diversity and molecular complexity. In contrast to traditional transcriptome sequencing, scRNA-seq can quantify the characteristics of individual cells and has gradually become an important tool for assessing tumor heterogeneity, revealing cell interactions, and reconstructing the evolutionary lineage. Nonetheless, the comprehensive characterization of iCCA at single-cell resolution remains rare. In this study, we included 4,239 high-quality cells from 10 iCCA samples and further analyzed cell composition and tumor heterogeneity, which represented the whole tumor ecosystem of iCCA. Meanwhile, we discovered that proteasome-related genes were unbalanced in the pseudo-time trajectory analysis, which indicated that the proteasome possibly played a key role in iCCA oncogenesis and development. Data collected from TCGA also demonstrated that iCCA tissues have higher expression levels of proteasome-related genes than normal tissue samples, including PSMA3 and PSMC2, which have been shown to promote the progression of biliary cancers (Verathamjamras et al., 2017; Duan et al., 2021; Zhu et al., 2021). These bioinformatic analyses revealed that targeting proteasomal activity could be a promising therapeutic approach.

Bortezomib, a first-generation proteasome inhibitor, induces endoplasmic reticulum stress and apoptosis in CCA cells (Vaeteewoottacharn et al., 2013). Drug screening analysis utilizing PDX-derived cells also revealed that proteasome inhibitors have an inspiring curative effect, especially in PTEN-deficient CCA cells (Jiang et al., 2020). However, the clinical therapeutic outcomes of bortezomib, in a single-agent trial of advanced CCA, have been disappointing (Denlinger et al., 2014). The discrepancy in bortezomib treatment of CCA *in vitro* and *in vivo* may be due to drug metabolism and the complexity of the tumor microenvironment. Compared with bortezomib, the next-generation proteasome inhibitor MLN2238, also named ixazomib, has better pharmacodynamics, pharmacokinetics, and anti-cancer activity (Kupperman et al., 2010; Muz et al., 2016). Our work is the first to demonstrate the therapeutic effect of MLN2238 in iCCA. Consistent with the results of previous studies on other solid tumors, our preclinical findings indicated that MLN2238 suppressed proliferation, blocked the cell cycle in the G2/M phase, and induced apoptosis in iCCA cells (Engür and Dikmen, 2017; Liu et al., 2017; Augello et al., 2018). We believe that MLN2238 may be a potential therapeutic choice for iCCA.

Autophagy is a process in which endogenous proteins and dysfunctional organelles are enclosed and delivered to the lysosomes for degradation. In the progression of tumor development, autophagy usually plays a dual role: on the one hand, autophagy can provide raw materials by reusing cytoplasmic macromolecules and damaged organelles for cell regeneration; on the other hand, excessive autophagy induces autophagic cell death. In addition to autophagy, the ubiquitin-proteasome system is the main pathway involved in the degradation of intracellular proteins. Several studies have revealed that inhibition of proteasomal activity induces cytoprotective autophagy (Zhu et al., 2010; Zang et al., 2012). However, recent research has also reported that proteasome inhibitors block autophagy flux to enhance cell apoptosis (Kao et al., 2014). These conflicting results may be ascribed to the dissimilar chemical structures of proteasome inhibitors and different types of cancer cells. For the first time, we uncovered the autophagy-induced effect of MLN2238 using immunofluorescence, western blotting, and detection of autophagy flux. In addition, the proteasome inhibitor (MLN2238) and the autophagy inhibitor (CQ) exerted a synergistic proapoptotic effect in iCCA cells. Nonetheless, the exact mechanism through which MLN2238 induces autophagy remains unclear and requires further investigation.

**FIGURE 9:**
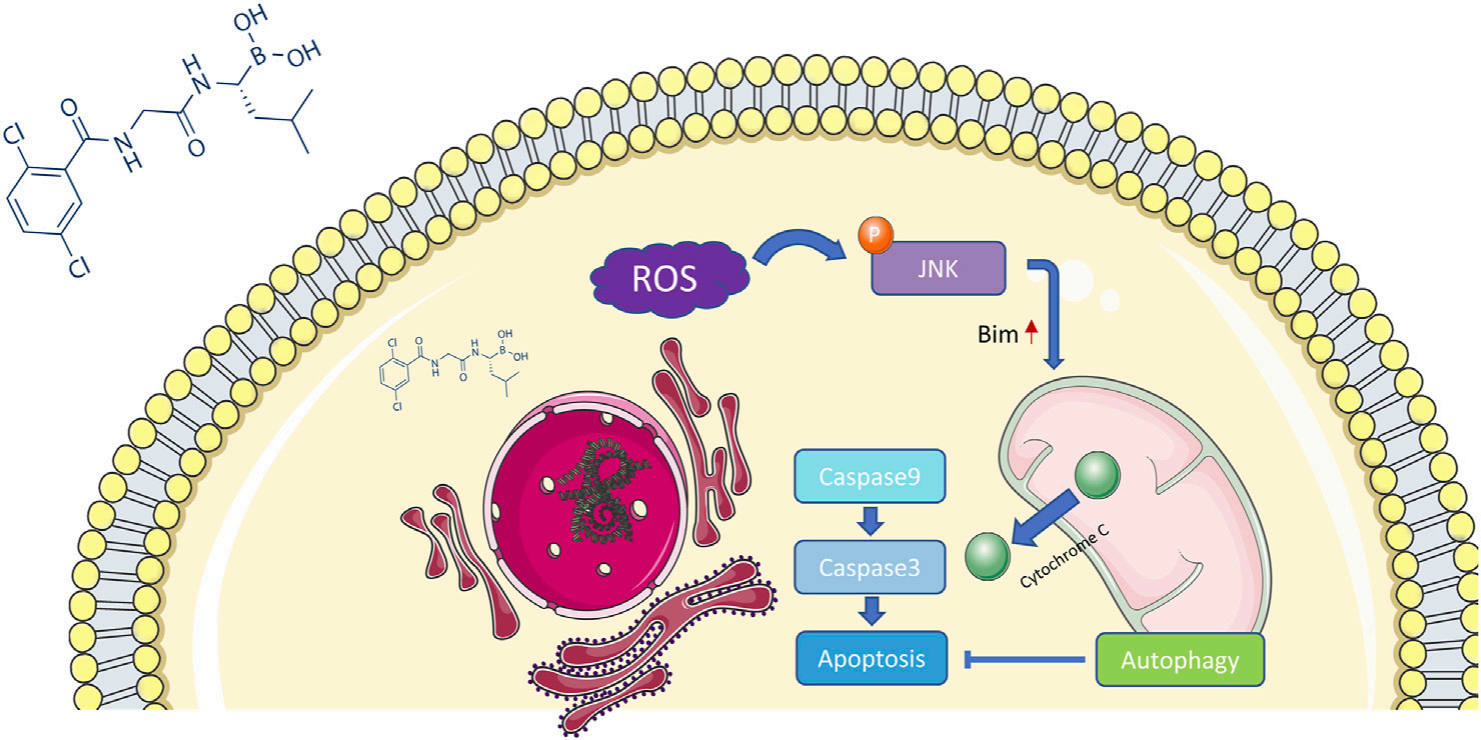
Potential mechanism of MLN2238 inducing apoptosis in iCCA.

Previous studies have demonstrated that proteasome inhibition can influence the degradation of unfolded proteins, which accumulate to trigger endoplasmic reticulum stress (Lee et al., 2003; Obeng et al., 2006). Unresolved endoplasmic reticulum stress generates a large amount of ROS and further induces oxidative stress, which in turn enhances endoplasmic reticulum stress (Lipchick et al., 2016). JNK is a member of the MAPK family and can be activated by ROS, actin cytoskeleton alterations, and pro-inflammatory cytokine stimulation (La Marca and Richardson, 2020). In this study, we discovered that MLN2238 increased intracellular ROS levels and activated the JNK signaling pathway in iCCA cells. Furthermore, apoptosis rates and JNK activation could be reversed by the ROS scavenger NAC, indicating that the ROS/JNK signaling pathway plays an important role in MLN2238-induced apoptosis. However, JNK has two effects on cancer and can mediate apoptosis or cellular transformation depending on different mechanisms (Tournier, 2013). Controversially, several studies have reported that the proapoptotic effect of proteasome inhibitors is closely associated with the JNK signaling pathway, but some studies have shown that JNK mediates a cytoprotective response against proteasome inhibition (Yu et al., 2004; Yin et al., 2005; Wang et al., 2009; Zanotto-Filho et al., 2012; Selimovic et al., 2013). In our study, suppression of JNK activation by SP600125 significantly reversed MLN2238-induced apoptosis and reduced mitochondrial membrane potential. Consequently, the proteasome inhibitor MLN2238 possibly induced apoptosis in iCCA cells *via* the ROS/JNK/mitochondrial pathway (Figure 9).

As a BH-3 only protein, Bim induces apoptosis under stress stimuli (Puthalakath et al., 2007). Previous studies have proved that Bim could act as a connection point linking endoplasmic reticulum stress and apoptosis (Soo et al., 2012). The occurring of endoplasmic reticulum stress could lead to the upregulation of Bim and then triggers the Bim-dependent apoptosis in multiple cell types including cancer cell lines (Puthalakath et al., 2007). It has also been demonstrated that Bim is the downstream of endoplasmic reticulum stress in pancreatic islet under glucotoxicity condition (Wali et al., 2014). As is mentioned above, proteasome inhibition is a major cause of endoplasmic reticulum stress, indicating proteasome inhibitors may kill cancer cells through endoplasmic reticulum stress and Bim-dependent apoptosis. A study in chronic myeloid leukemia showed that the administration of proteasome inhibitor oprozomib could induce cell apoptosis *via* ASK-JNK-Bim axis (Wang et al., 2021). Combination of bromodomain and extra-terminal domain (BET) protein inhibitor and proteasome inhibitor improved therapeutic outcome in patients with advanced multiple myeloma by promoting endoplasmic reticulum stress and Bim-dependent apoptosis (Qian et al., 2018). In this study, the upregulation of Bim was observed, indicating Bim may participate in the mechanism which accounts for the iCCA cell death caused by MLN2238.

Recently, tumor microenvironment has been regarded as a therapeutic target in cancers (Xiao and Yu 2021). Apart from targeting malignant cells, proteasome inhibitors have also been found to influence tumor microenvironment. It has been reported that bortezomib, a first-generation proteasome inhibitor, could regulate the tumor microenvironment of head and neck cancer by recruiting tumor infiltrating immunocytes including CD4^+^/CD8^+^ T cells, B cells, macrophages and NK cells (Benvenuto et al., 2021). While increasing the number of infiltrating immune cells in tumor microenvironment, bortezomib also displayed ability of enhancing the antitumor function of CD8^+^ T cells through miR-155-SOCS1/SHIP1-T-bet-PD1 axis (Renrick et al., 2021). However, except for bortezomib, there is a lack of knowledge about effects of other proteasome inhibitors on tumor microenvironment. Besides, researches on mechanisms underlying the effects of proteasome inhibitors on tumor microenvironment are insufficient. The potential relationship between proteasome inhibitors and tumor microenvironment may become a novel research topic in the future. Explorations on such aspect could largely expand the clinical application of proteasome inhibitors since the development of medication alignments which target malignant cells and tumor microenvironment simultaneously may emerge as a new therapeutic option (Wu and Dai 2017).

To better simulate the tumor microenvironment, we further explored the effects of MLN2238 in an iCCA tumor-bearing mouse model. Consistent with the *in vitro* data, MLN2238 significantly inhibited the growth of iCCA xenografts and decreased the expression of ki-67. Immunohistochemistry and western blotting also demonstrated the activation of the JNK signaling pathway. However, the blood test results showed increasing levels of TBA and BUN after treatment with high concentrations of MLN2238, which indicated that the drug probably possessed a certain degree of hepatotoxicity and nephrotoxicity. Therefore, using nanotechnology to improve tumor targeting and biosafety is the next direction of our research.

## 5 Conclusion

For the first time, our work elucidated proteasome-related genes, using scRNA-seq analysis, that were significantly out of balance in iCCA progression. According to TCGA database, iCCA tumor tissues had higher expression levels of proteasome-related genes than normal tissues. MLN2238, a proteasome inhibitor, suppresses proliferation, blocks the cell cycle in the G2/M phase, promotes apoptosis, and induces cytoprotective autophagy in iCCA cells. The ROS/JNK/mitochondrial signaling pathway may play an essential role in MLN2238-induced apoptosis. Moreover, MLN2238 inhibited tumor growth and activated the JNK pathway *in vivo*. This study revealed that targeting the proteasome could be a promising therapeutic method for iCCA.

## Data Availability

The original contributions presented in the study are included in the article/Supplementary Material, further inquiries can be directed to the corresponding authors.
