# Supplementary figures and images for: MLN2238 exerts its anti-tumor effects via regulating ROS/JNK/mitochondrial signaling pathways in intrahepatic cholangiocarcinoma
Source: Front Pharmacol. 2022 Oct 31;13:1040847. doi: 10.3389/fphar.2022.1040847 (PMC9659592; doi:10.3389/fphar.2022.1040847)

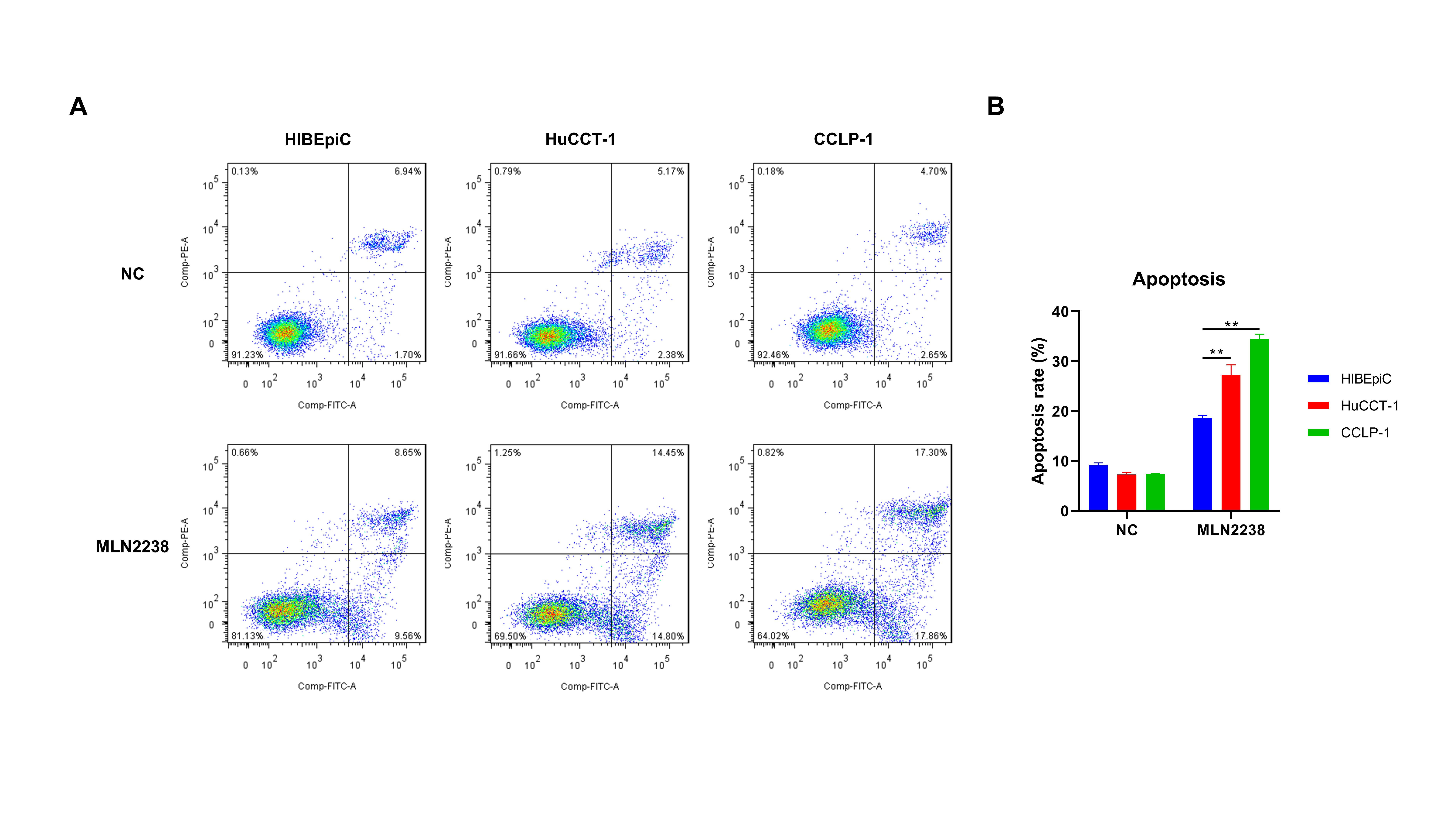

Supplement: Supplementary file 1 [file Image6.TIF]

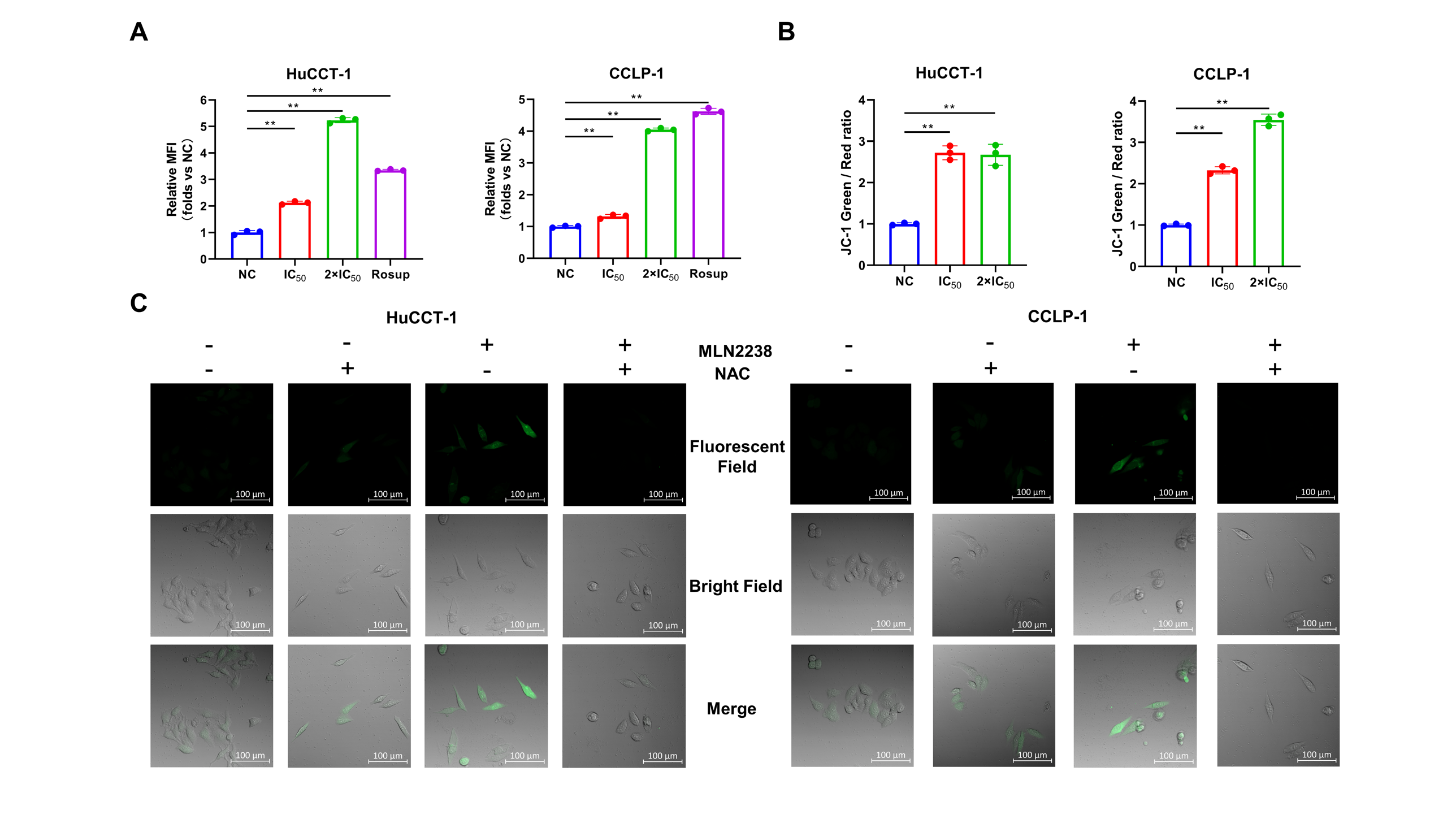

Supplement: Supplementary file 3 [file Image3.TIF]

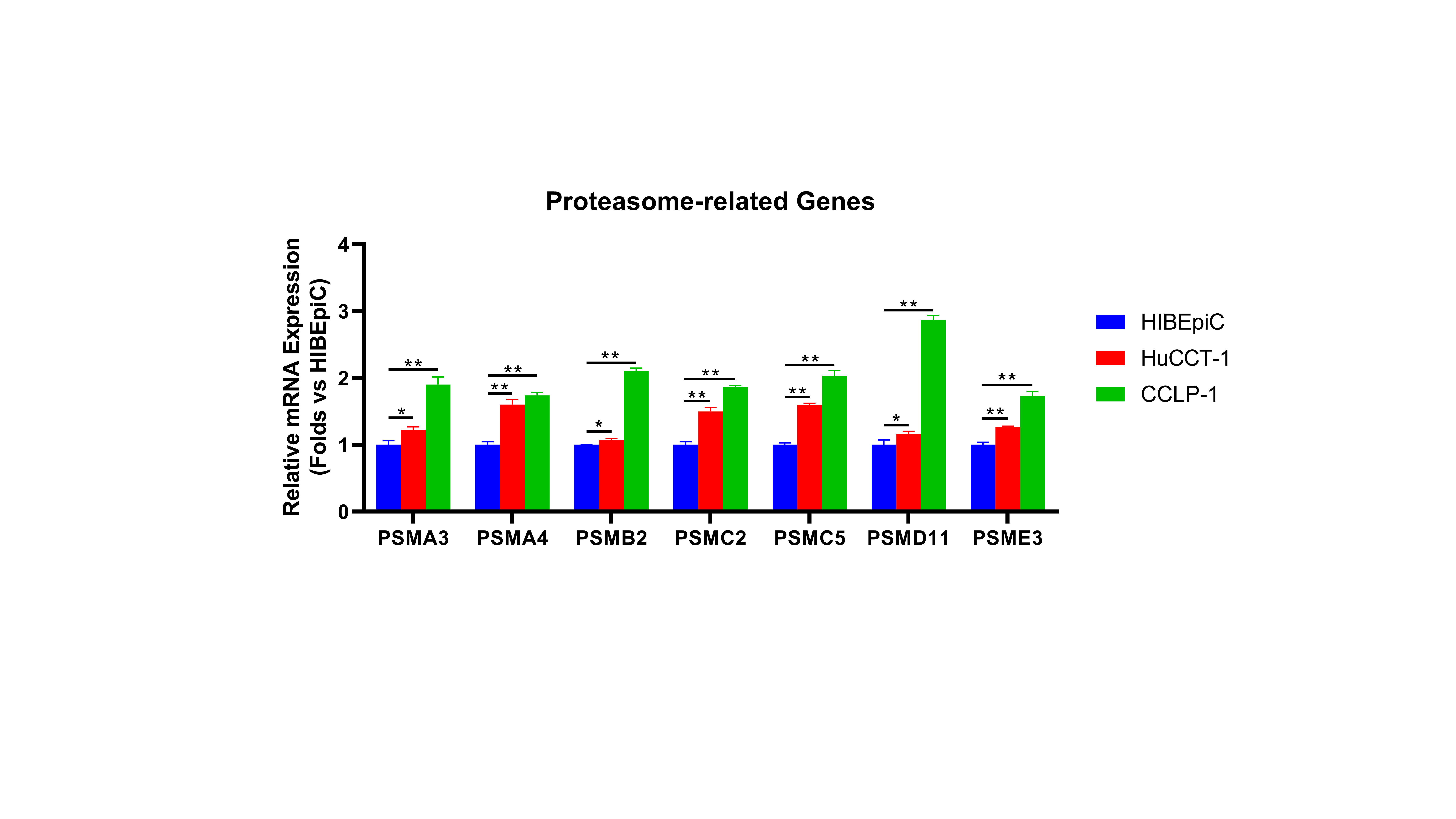

Supplement: Supplementary file 4 [file Image4.TIF]

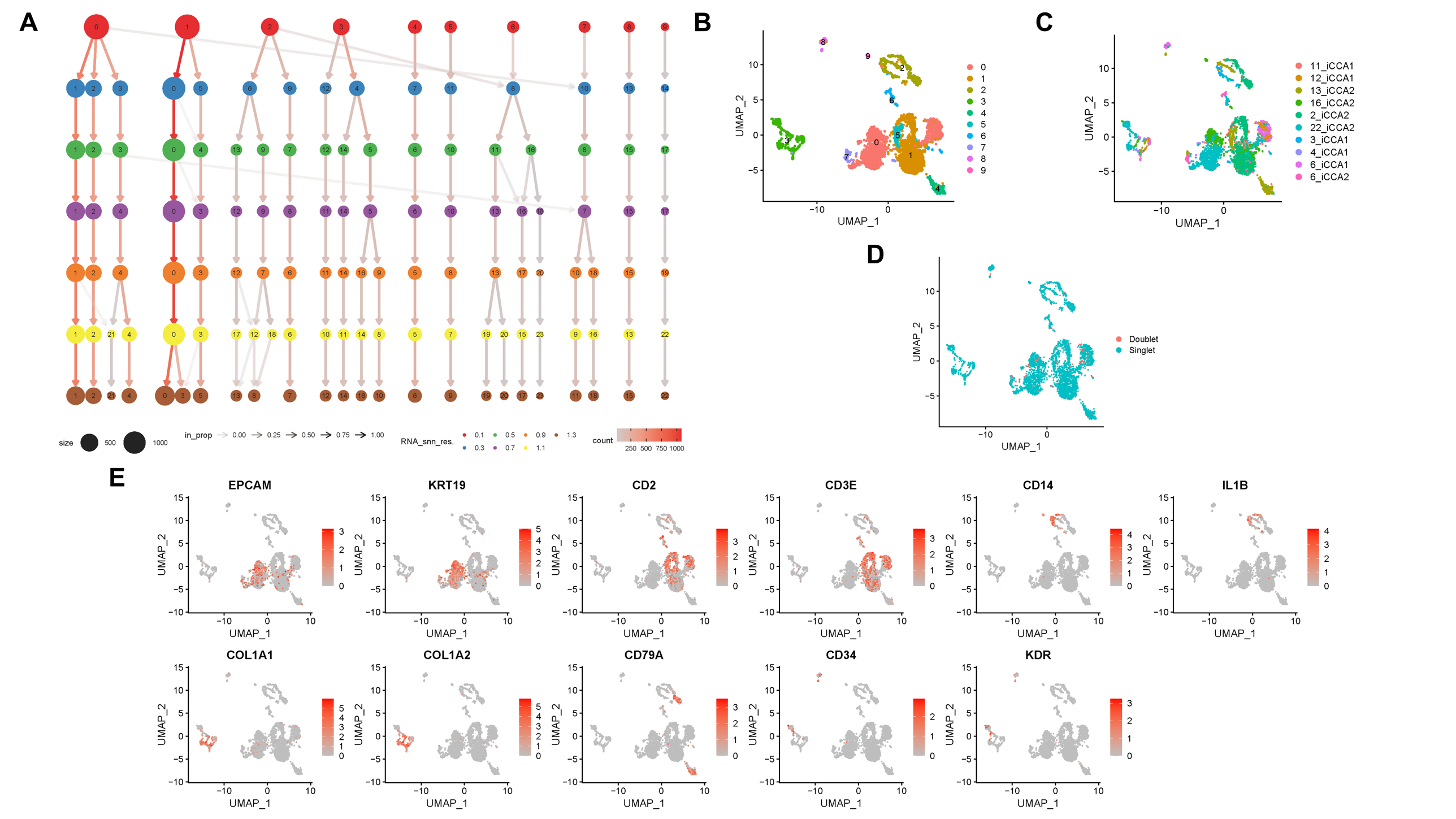

Supplement: Supplementary file 5 [file Image2.TIF]

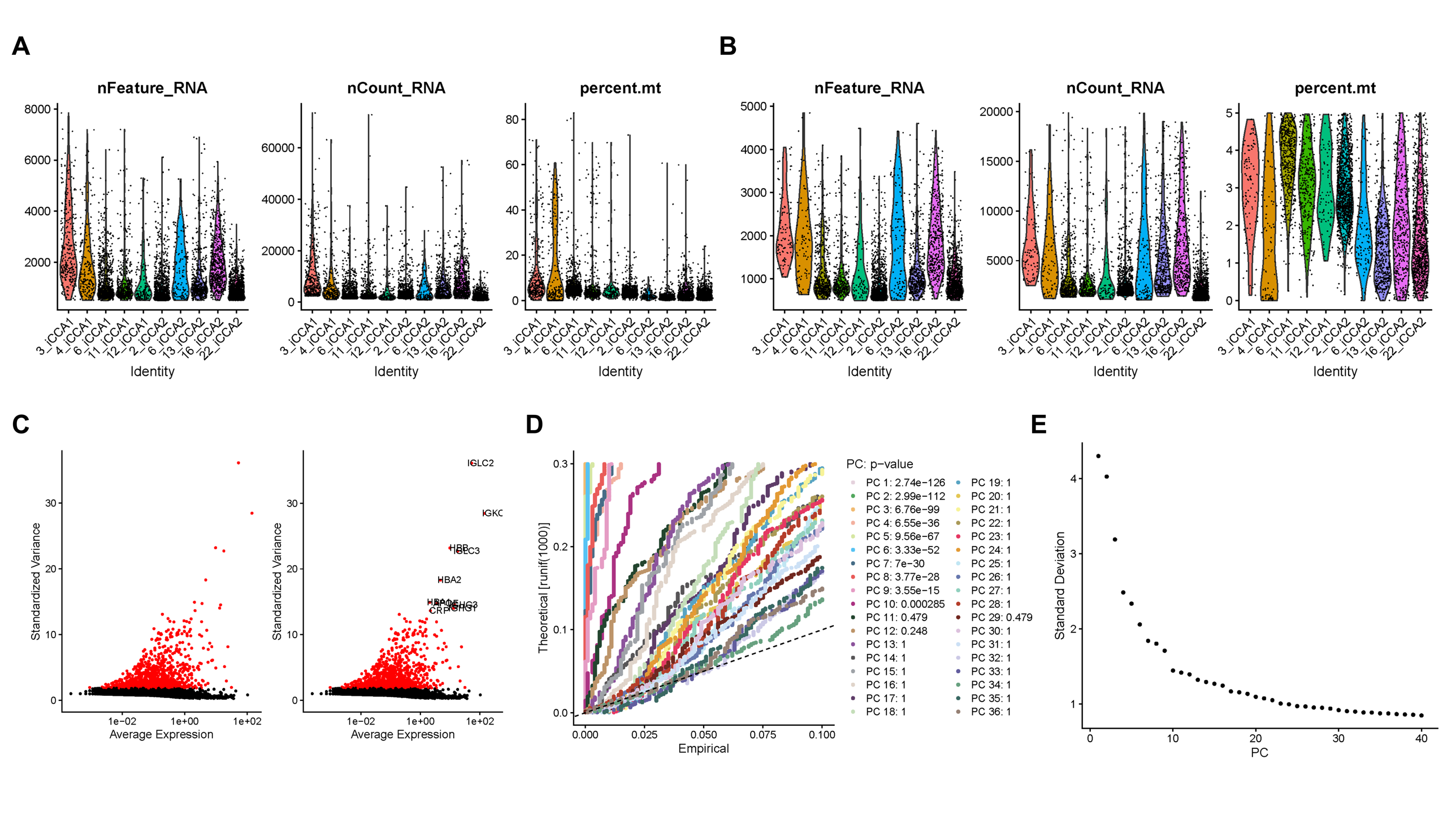

Supplement: Supplementary file 6 [file Image1.TIF]

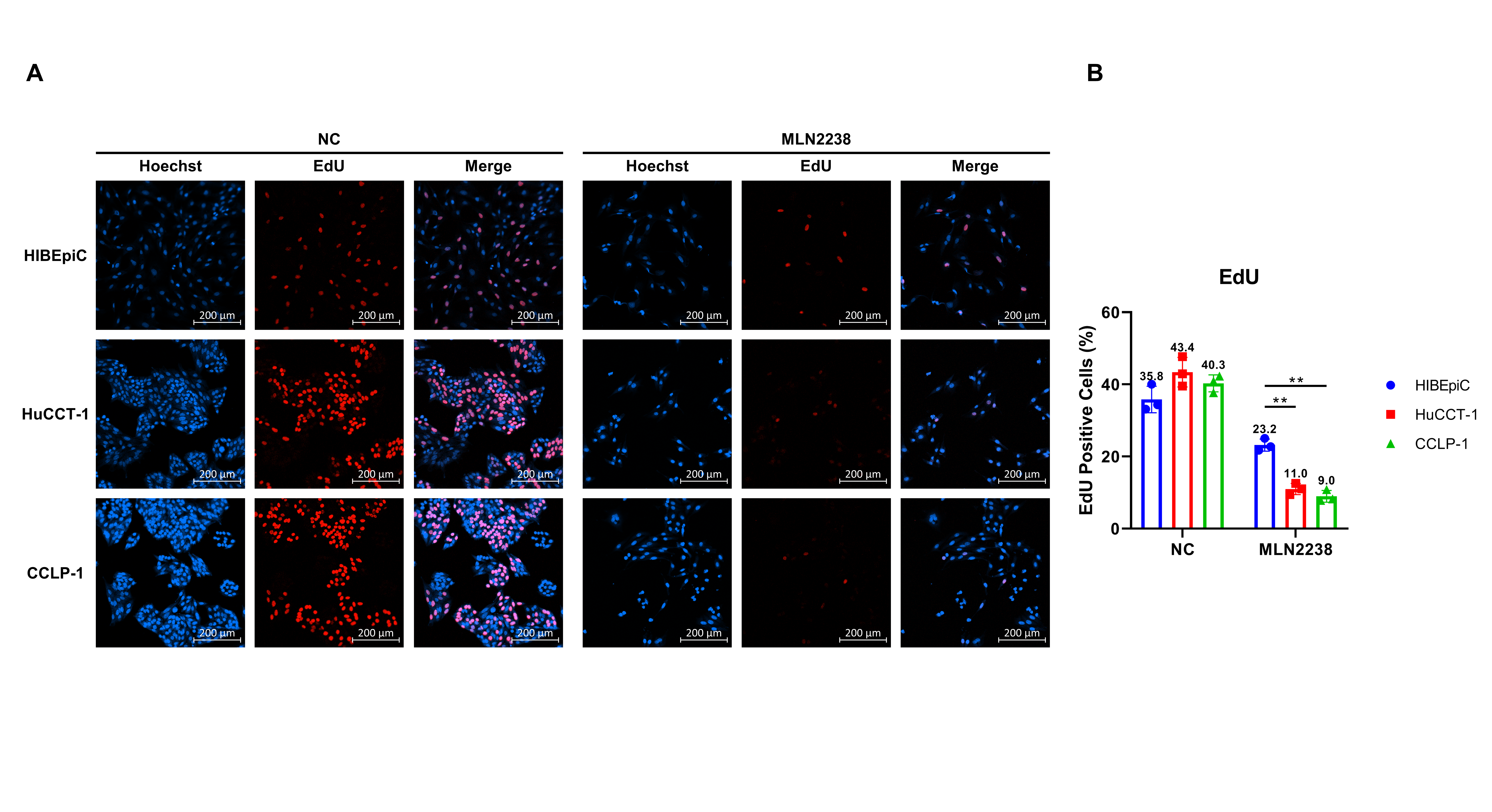

Supplement: Supplementary file 7 [file Image5.TIF]
