# Supplementary material for: MLN2238 exerts its anti-tumor effects via regulating ROS/JNK/mitochondrial signaling pathways in intrahepatic cholangiocarcinoma
Source: Front Pharmacol. 2022 Oct 31;13:1040847. doi: 10.3389/fphar.2022.1040847 (PMC9659592; doi:10.3389/fphar.2022.1040847)
Supplement: Supplementary file 2 [file Table1.DOCX]

**Primer sequences used for quantitative real-time PCR**

| Gene | Forward primer  (5’→3’) | Reverse primer  (5’→3’) |
| --- | --- | --- |
| PSMA3 | GCTCAATCGGCACTGGGTAT | ACCTGCTACTGCCATTCCAAC |
| PSMA4 | GGAGCCAATACCTTGTGAGCA | GCAATGAAACACCAAAGGGACG |
| PSMB2 | ATCCTCGACCGATACTACACAC | GAACACTGAAGGTTGGCAGAT |
| PSMC2 | GAGCACTTACTCTAGGCAGATCA | GTACACCTGGCAACCTGTAAAG |
| PSMC5 | AGGCACAGAGGAACGAACTAA | AGGATGTACCTTGACCAACACTT |
| PSMD11 | TCGCCTGGTCCGATCTCTT | ATGCACTCTAAACACAGCTCG |
| PSME3 | AAGGTTGATTCTTTCAGGGAGC | AGTGGATCTGAGTTAGGTCATGG |
| GAPDH | AACGGATTTGGTCGTATTGGG | CCTGGAAGATGGTGATGGGAT |
